# Applications of injectable hemostatic materials in wound healing: principles, strategies, performance requirements, and future perspectives

**DOI:** 10.7150/thno.86930

**Published:** 2023-08-21

**Authors:** Hong Wang, Liang Yang

**Affiliations:** School of Physics and Electronic Information, Yan'an University, Yan'an, 716000, China.

**Keywords:** Hemostasis, Platelet, Fibrinogen, Injectable, Synthetic strategy

## Abstract

Uncontrolled traumatic bleeding can lead to death due to excessive blood loss within minutes. Early intervention is crucial to save lives, making timely and effective hemostasis is a major global challenge. Injectable hemostatic materials (IHMs) have been proposed to improve the effectiveness of hemostasis, facilitate wound healing, and enhance survival rates in emergency situations. The superior hemostatic performance of IHMs has garnered significant attention. However, there are relatively few comprehensive reviews on IHMs. This paper aims to provide a comprehensive review of the latest research progress on IHMs in recent years. Firstly, the physiological hemostatic process and the underlying principles of hemostasis are analyzed. Subsequently, the synthesis strategies for different IHMs are discussed. The performance requirements of IHMs are then summarized, including high efficiency, biocompatibility, degradability, manipulability, stability and antibacterial ability. Finally, the development prospects and challenges of IHMs are presented. This review serves as a necessary and systematic summary of IHMs, providing a valuable reference for the development of new high-performance hemostatic materials and their practical clinical applications.

## 1. Introduction

**Figure 1:**
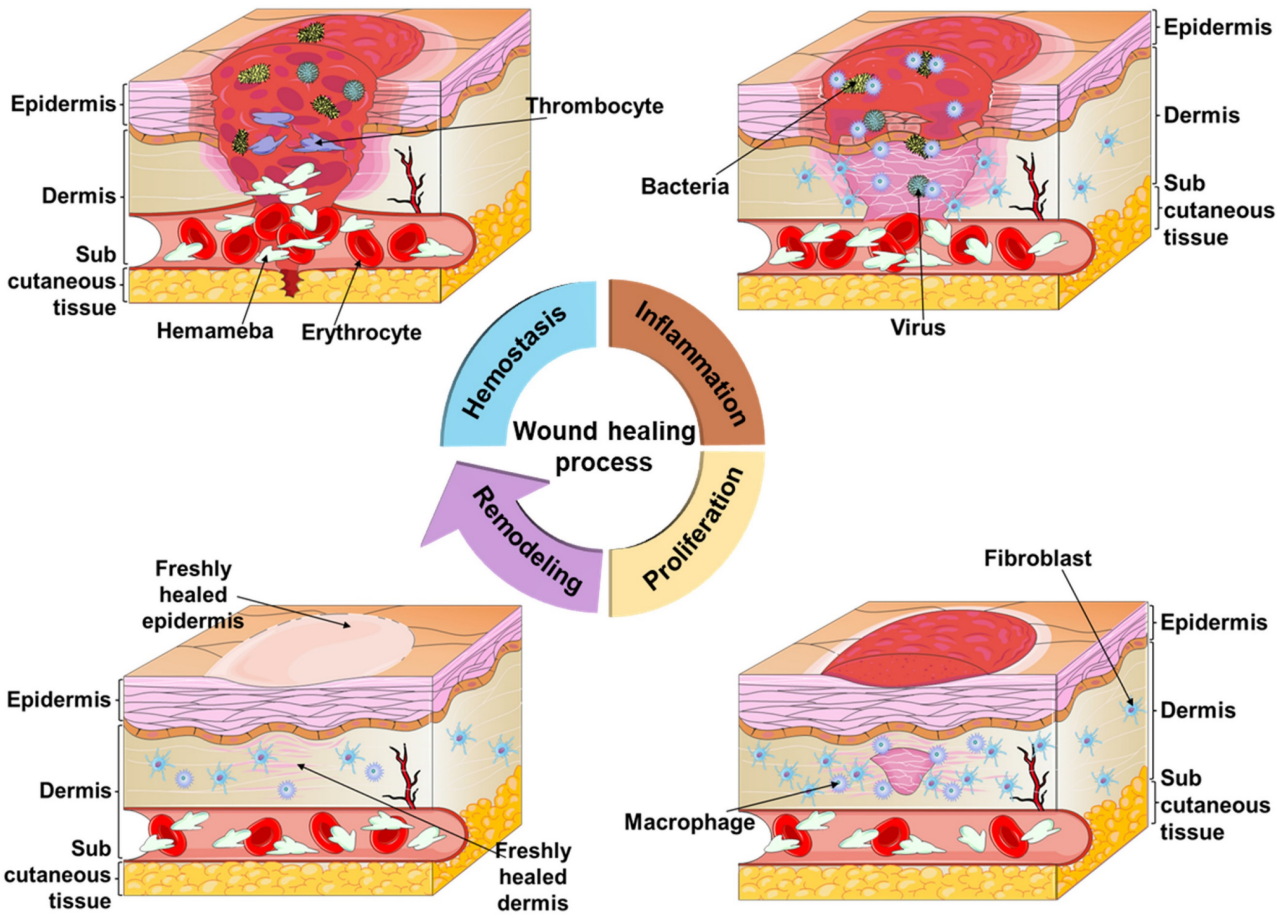
Diagram of the wound healing process, which consists of four phases, namely, hemostasis, inflammation, proliferation and remodeling. During the hemostasis phase, the body's emergency repair system and blood clotting system are activated. In the inflammation phase, the focus is on wound debridement by removing phagocytic debris and bacteria, and this phase usually lasts four to six days. The proliferation occurs during the first few weeks of wound healing, and the main focus is on filling and covering the wound. The final remodeling phase, in which the new tissue slowly gains strength and flexibility, is more prolonged and usually lasts from 21 days to one year. Figures created with scifig.com.

The skin, the largest organ of the body, is the tissue that wraps around the muscles and covers the entire body's surface. Its primary functions include protecting the body, regulating body temperature, sensing external stimuli, perspiration, secretion, and defense against foreign pathogens 1, 2. It serves as a protective barrier against physical, mechanical, chemical, and microbial attacks, safeguarding various tissues and organs. When the skin is injured, wound healing takes place in four phases: hemostasis, inflammation, proliferation, and remodeling 3-6, as shown in Figure 1. Hemostasis involves a complex physiological process where a blood clot forms 7, blocking the broken blood vessels and preventing excessive bleeding. However, in case of severe bleeding, relying solely on the body's natural hemostatic mechanisms might not be sufficient, necessitating hemostatic interventions for efficient bleeding control 8, 9. The uncontrollable traumatic bleeding becomes a serious medical emergency 10, 11 and is a major cause of death in accidents, military conflicts, natural disasters, and traffic incidents for both civilians and military personnel 9, 12-14. Given that most deaths occur within the first 60 minutes after trauma, timely bleeding control is crucial, and effective measures within this timeframe are essential for improving survival rates and reducing associated complications, such as excessive bleeding 13, 15-18.

Furthermore, several diseases or surgical potentialities can increase the risk of bleeding, turning it into a potentially dangerous situation that needs to be addressed. Hemostatic materials play a crucial role in wound treatment, especially in severe cases where the original hemostatic system is unable to control the bleeding. Early intervention with hemostatic materials at specific sites to prevent further worsening can be a potential approach. The effectiveness and benefits of existing hemostatic materials in various environments have been demonstrated 19. However, there are still significant challenges in developing cost-effective, safe, effective, and easy-to-use hemostatic materials, such as high production costs, complex manufacturing processes, and limited scalability. The main hemostatic materials currently used for bleeding control are topical hemostatic materials and injectable hemostatic materials (IHMs). The former is mainly used for external compressible bleeding 15, while the latter is a new type of hemostatic material developed based on the limitations of topical hemostatic materials in the treatment of incompressible wounds with internal bleeding 20-24, which has seen great innovation in the treatment of internal bleeding in recent years. Platelet- and fibrin-based IHMs offer unique advantages, including activation of the coagulation cascade and acceleration of coagulation, making them highly valuable for research and application in hemostasis.

**Figure 2:**
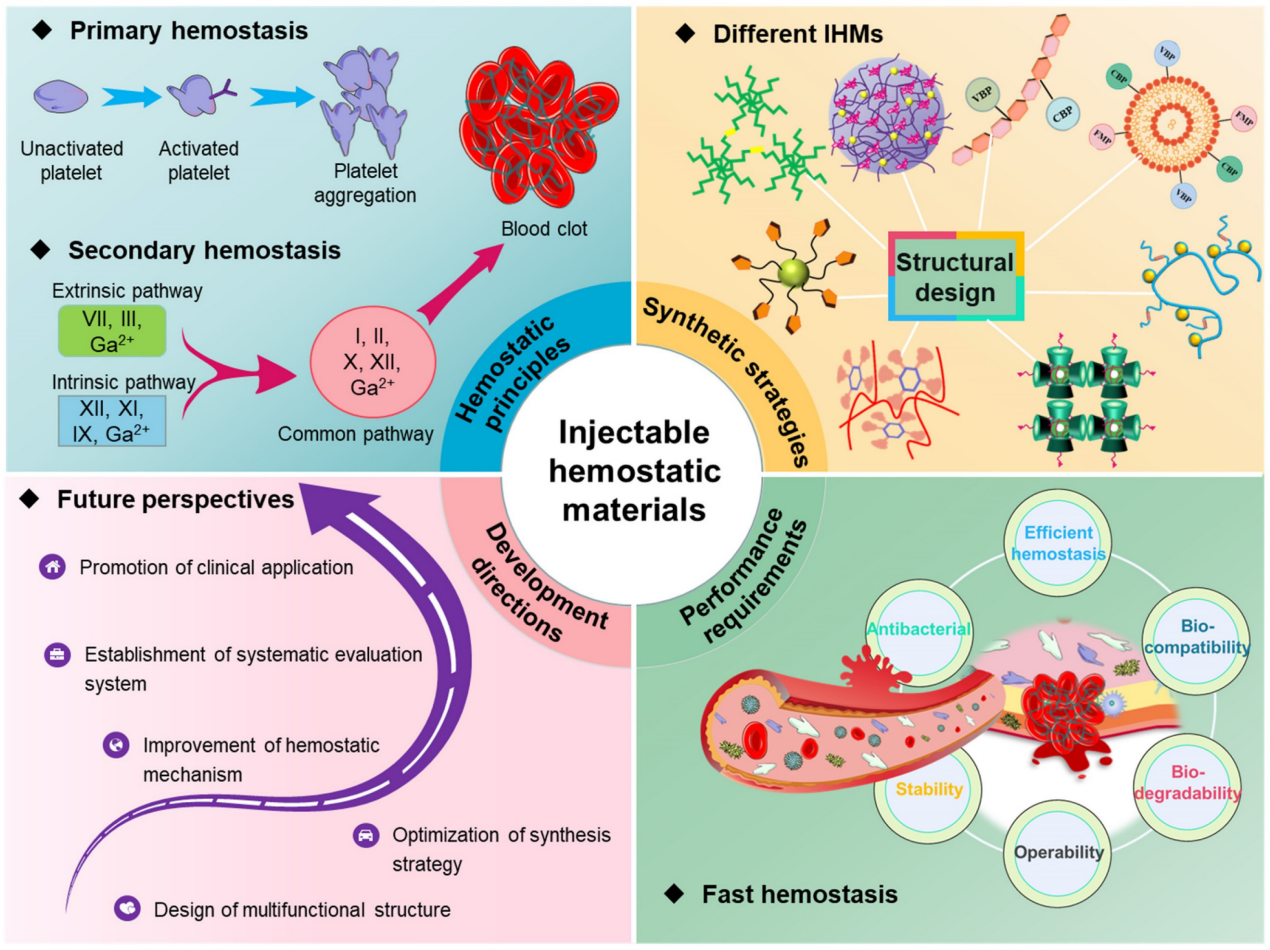
Overall overview of IHMs, focusing on the summary and analysis of hemostatic principles, synthesis strategies, performance requirements, and development directions, which correspond to the chapter layout of this review.

Currently, researchers have tried and explored several studies on IHMs, and some progress has been made in rapid hemostasis. As shown in Figure 2, this paper firstly provides a systematic analysis of the hemostatic principles, then analyses the synthesis strategies and technologies for different hemostatic materials, summarizes the performance requirements of IHMs developed based on hemostatic characteristics, and finally prospects the potential future development directions, which lays the foundation for the development and application of hemostatic materials.

## 2. Hemostatic principles

Understanding the hemostatic mechanism of materials, particularly the interactions with blood cells, platelets, and other blood components, is crucial for effective hemostasis. By gaining a deeper understanding of these materials' mechanisms and the appropriate timing for their use, hemostatic surgery can be conducted more quickly and accurately to avoid treatment delays. Moreover, this understanding helps healthcare professionals predict potential complications and develop appropriate measures to address them. Therefore, comprehending the physiological processes and hemostatic mechanisms of IMHs is essential for enhancing healthcare professionals' treatment capabilities, as well as ensuring patient safety and well-being.

### 2.1 Physiological hemostasis

**Figure 3:**
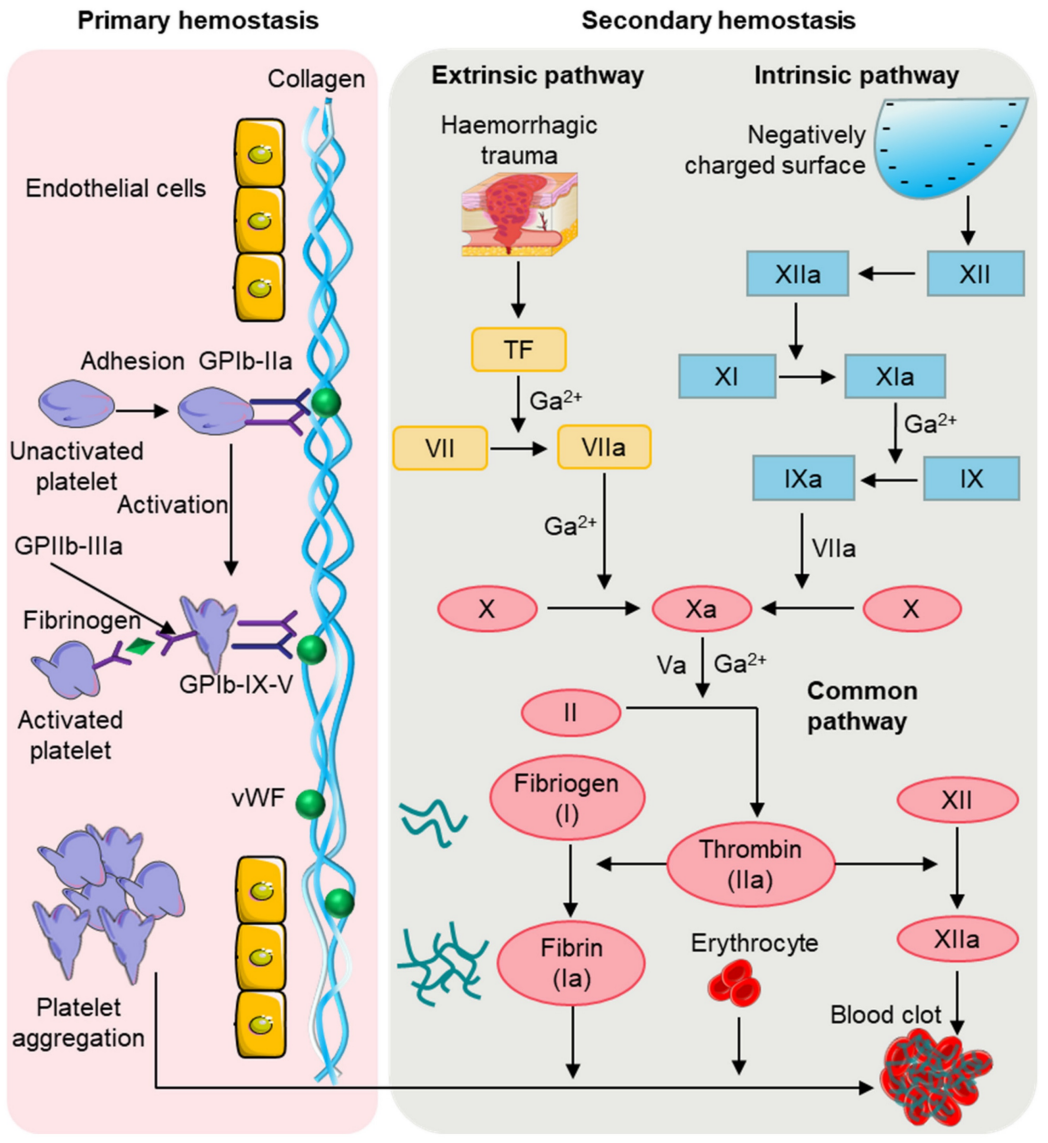
Physiological hemostasis process. In the primary stage of hemostasis, platelets adhere to the site of injury where they are activated. Activated platelets further activate and aggregate free platelets to form a platelet plug. In the second stage of hemostasis, thrombin, produced by the extrinsic and intrinsic coagulation pathways, converts fibrinogen to fibrin, which then forms a thrombus with the platelet plug and blood cells.

Hemostasis is a complex physiological process that involves the regulation of platelets, cells, plasma proteins, and clotting factors in the blood to achieve hemostasis 25. After an injury, the human body typically relies on physiological processes to achieve natural hemostasis, quickly stopping bleeding in blood vessels. This phenomenon, known as physiological hemostasis, is an essential protective mechanism of the body. When blood vessels are damaged, the rapid formation of a blood clot is required to prevent blood loss, while simultaneously limiting the hemostatic response to the site of injury to maintain the fluid state of blood inside the body's blood vessels. As depicted in Figure 3, physiological hemostasis mainly involves two stages 26: the first stage primarily includes vasoconstriction and platelet thrombus formation, while the second stage mainly refers to blood clotting. Thus, physiological hemostasis is the result of multiple factors and mechanisms working together to maintain a delicate balance. When the function of physiological hemostasis is weakened, there can be a tendency for bleeding and the occurrence of bleeding disorders. Conversely, excessive activation of physiological hemostasis can lead to pathological blood clot formation.

When bleeding occurs, local and nearby small blood vessels of the damaged blood vessels contract, reducing local blood flow, which is conducive to reducing or preventing bleeding 27. The causes of vasoconstriction can be mainly attributed to the following three aspects: firstly, injury stimulation reflex leads to vasoconstriction; secondly, damage to the vascular wall causes local vascular myogenic contraction; thirdly, the platelets attached to the injury release substances such as 5-HT and TXA₂, causing vasoconstriction. Platelets are usually inactive, but when the skin is exposed, a small number of platelets adhere to the collagen. Through the adhesion of platelets, the injured site can be "identified" so that the thrombus can be correctly located. The attached platelets further activate intracellular signaling pathways, leading to platelet activation and the release of endogenous adenosine diphosphate (ADP) and thromboxane A2 TXA_2_ 28. This activation triggers other platelets in the blood to aggregate and adhere to each other, resulting in irreversible aggregation. Both the ADP released by locally damaged red blood cells and the thrombin generated in the process of local coagulation can make the platelets flowing near the wound constantly stick to and gather on the platelets that have been attached and fixed on the inner subcutaneous collagen. This eventually form a platelet plug to stop thrombosis and clog the wound, thus achieving preliminary hemostasis, which is the first stage of hemostasis. In the process of hemostasis, platelets quickly respond to the injured site through adhesion, release, aggregation, contraction, and activation 29.

In the second stage of hemostasis, the coagulation system is activated, leading to rapid local blood coagulation, converting soluble fibrinogen in the plasma into insoluble fibrin 30, which interweaves to reinforce the blood clot. The coagulation process involves the intrinsic pathway, extrinsic pathway, and common pathway. The coagulation factors involved in the intrinsic pathway mainly include XII, XI, and IX 31. Factor XII is initially activated into factor XIIa, which then activates factor XI to form factor XIa, followed by factor XIa promoting factor IX to form factor IXa. Factor IXa then binds with factor VIIIa to form the prothrombinase complex, which converts factor X to factor Xa. In contrast to the intrinsic pathway, not all the coagulation factors involved in the extrinsic pathway come from the blood 32, 33. The extrinsic pathway is initiated by the exposure of transmembrane glycoprotein tissue factor and mainly involves factors III and VII 34. In the presence of Ca^2+^, the tissue factor and factor VII synergistically form the VIIa complex, which then activates factor X to form factor Xa. Eventually, the intrinsic and extrinsic pathways combine to form a common pathway. In the presence of Ca^2+^ and phospholipid membranes, factor Xa and factor Va interact to form the prothrombinase complex, and thrombin converts fibrinogen into fibrin, forming fibrin monomers 35. Additionally, thrombin activates factor XIII to form factor XIIIa, which, with the combined action of Ca^2+^ and fibrin, promotes the formation of a dense blood clot for permanent hemostasis.

Although physiological hemostasis is divided into two hemostatic processes, they occur sequentially and overlap with each other, closely interconnected. Platelet adhesion is facilitated when vasoconstriction slows down blood flow, and the release of 5-HT and TXA_2_ during platelet activation further promotes vasoconstriction 28. Activated platelets provide a phospholipid surface that activates coagulation factors during blood coagulation. Platelets can bind various coagulation factors on their surface and also release fibrinogen and other coagulation factors, thereby greatly accelerating the clotting process 34. And the thrombin that produces in blood clotting process can strengthen platelet activation again. In addition, the contraction of platelets within a blood clot can cause the clot to retract, squeezing out the serum and making the clot more solid, effectively sealing the vessel puncture. Therefore, the two processes of physiological hemostasis mutually reinforce each other, ensuring timely and rapid hemostasis, with platelets playing a very important role in this process. In the presence of thrombocytopenia or reduced platelet function, the bleeding time is prolonged.

### 2.2 Analysis of key factors of hemostasis

To achieve effective and accelerated hemostasis, a variety of hemostatic materials have been developed that regulate the above-mentioned physiological hemostatic processes through hemostatic mechanisms involving platelets, red blood cells, thrombin, fibrin and Ca^2+^ ions 19. These materials have become a particular focus of interest.

#### 2.2.1 Platelets and red cells

Platelet activation and aggregation are significant contributors to physiological hemostasis 36. Platelets release chemicals such as 5-HT and TXA_2_ during activation, which enhance vasoconstriction and reduce blood flow to the site of injury. Furthermore, activated platelets release clotting factors, including fibrinogen and Factor V, which speed up the clotting process. This eventually leads to the formation of platelet plugs that help staunch the bleeding. Numerous hemostatic materials utilizing platelets have been developed, such as nano-platelet bodies and platelet substitutes 37-39. These materials mimic platelet adhesion and aggregation, exhibiting enhanced hemostatic effect and effectively controlling severe bleeding. Erythrocytes also play a key role in hemostasis 40, which can enhance platelet adhesion and aggregation by releasing ADP and TXA_2_, and can also interact with fibrinogen to affect the structure and properties of clots 41, 42.

#### 2.2.2 Thrombin and fibrin

Thrombin is an enzymatic protein formed through enzymatic reaction of prothrombin. Prothrombin itself cannot directly play a hemostatic effect but is activated to produce thrombin, which has a hemostatic effect. When blood vessels are injured, tissue factors are released, allowing prothrombin and platelets in the plasma to interact with the factors. Through enzymatic reactions, prothrombin is activated, resulting in the formation of thrombin. Through enzymatic reactions, prothrombin is activated, leading to the formation of thrombin. Thrombin recognizes and accelerates the formation of fibrin strands, which stretch and form fibrin in the wound. These fibrin strands interweave and secure themselves around the ruptured blood vessel, forming a stable thrombus. Thus, thrombin and fibrin act synergistically. Thrombin stimulates the formation of fibrin, and when fibrin aggregates into a blood clot, the clot prevents blood from flowing out, thereby stopping bleeding, and facilitating wound repair. Fibrin and thrombin, as important components of coagulation, have been widely used 43, which can directly activate and accelerate the coagulation process, thus improving hemostatic efficacy 27, 44. However, some potential risks limit their application 45, 46, such as viral contamination and thrombosis. As demonstrated in Figure 3, many coagulation factors are involved in the formation of thrombin in the second stage of physiological hemostasis. Effectively stimulation of these coagulation factors to generate more thrombin and fibrin will be an important research direction to promote rapid hemostasis.

#### 2.2.3 Ca^2+^ ions

The Ca^2+^ ions play a very important role in the blood clotting process 47. As mentioned earlier, blood clotting is a physiological process that occurs in the body to stop bleeding. When a blood vessel ruptures, platelets initially block the wound, aggregate to form a thrombus, and begin to release certain substances that allow the body to further develop a hemostatic response 48. The Ca^2+^ ions are released and are involved in several physiological processes: (a) Ca^2+^ ions facilitate the formation of haemagglutinin. They aid in the formation of a coagulation complex involving factors Xa and Va, which is essential for blood clotting. (b) Ca^2+^ ions activate thrombinogen. They provide additional sites for the aggregation of the coagulation complex. (c) Ca^2+^ ions play a role in platelet activation. The protease is dependent on Ca^2+^ ions for activation. When platelets are stimulated, this protease is released, further promoting interplatelet aggregation and thrombus formation. (d) Ca^2+^ ions participate in the activation of various coagulation factors. They are involved in activating coagulation reactions and fibrin degradation reactions, thus promoting the formation of fibrinogen in the plasma. In summary, Ca^2+^ ions play a crucial role in the overall coagulation reaction, from the formation of enzymes to platelet activation.

## 3. Synthetic strategies of IHMs

**Figure 4:**
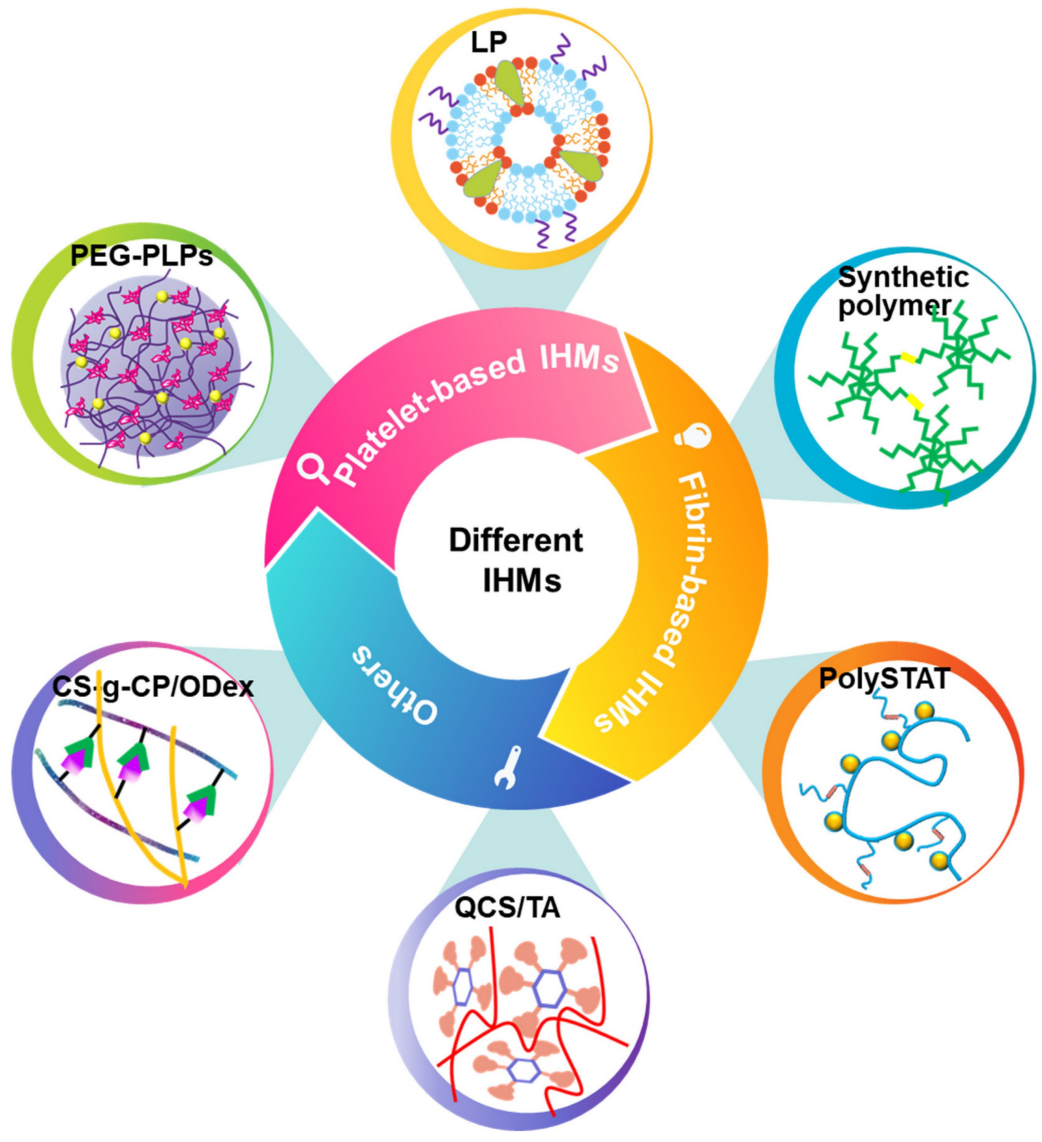
Classification and synthesis strategies of IHMs. IHMs can be classified into platelet-based IHMs, fibrin-based IHMs, and others according to the hemostatic principles. Different synthesis strategies can yield different IHMs, which usually have good hemostatic effects, mainly due to different structural design strategies.

Although researchers have developed many traditional hemostatic materials, they still have certain limitations in preventing bleeding quickly, such as treating invisible wounds. IHMs has been widely concerned for its ability to treat inaccessible wounds and has been considered as an effective strategy for the treatment of connected hemorrhages and battlefield casualties 49, 50. Although the research and development of IHMs faces severe challenges, researchers have made several attempts to obtain hemostatic materials with good donor supply, shelf life and refrigeration. The structural design of different materials makes an impact on the final performance as well 51-55. This section will summarize the recent research progress of these hemostatic materials, focusing on the synthesis strategy of IHMs, as shown in Figure 4.

### 3.1 Platelet-based IHMs

Immediate hemostatic interventions in people with severe bleeding are an important life-saving measure. As previously stated, platelets are an important component in preventing bleeding 56, 57 and their unique surface allows them to have immunological, adhesion, and activation effects 58, 59. Developing platelet-based IHMs is an effective and feasible strategy for achieving hemostasis. However, there are certain challenges associated with platelets, such as short shelf-life, insufficient supply, limited availability, and susceptibility to bacterial contamination 60-63. Several strategies have been proposed to address the short shelf life of platelets, such as ultra-low temperature preservation, use of platelet activation inhibitors, the addition of additives to platelet solutions, pathogen reduction techniques, and freeze-drying platelets 64, 65. Researchers have also developed techniques such as thromboplast 66 or covalent cross-linking 67 to stabilize the platelet properties of the lyophilized platelets. Thromboplast promotes clot and thrombin production, while covalent cross-linking is effective through cross-linking. Additionally, donor-independent techniques have been proposed to obtain safe and reliable platelets 68-70, thereby addressing concerns related to immune reactions and potential contamination risks.

**Figure 5:**
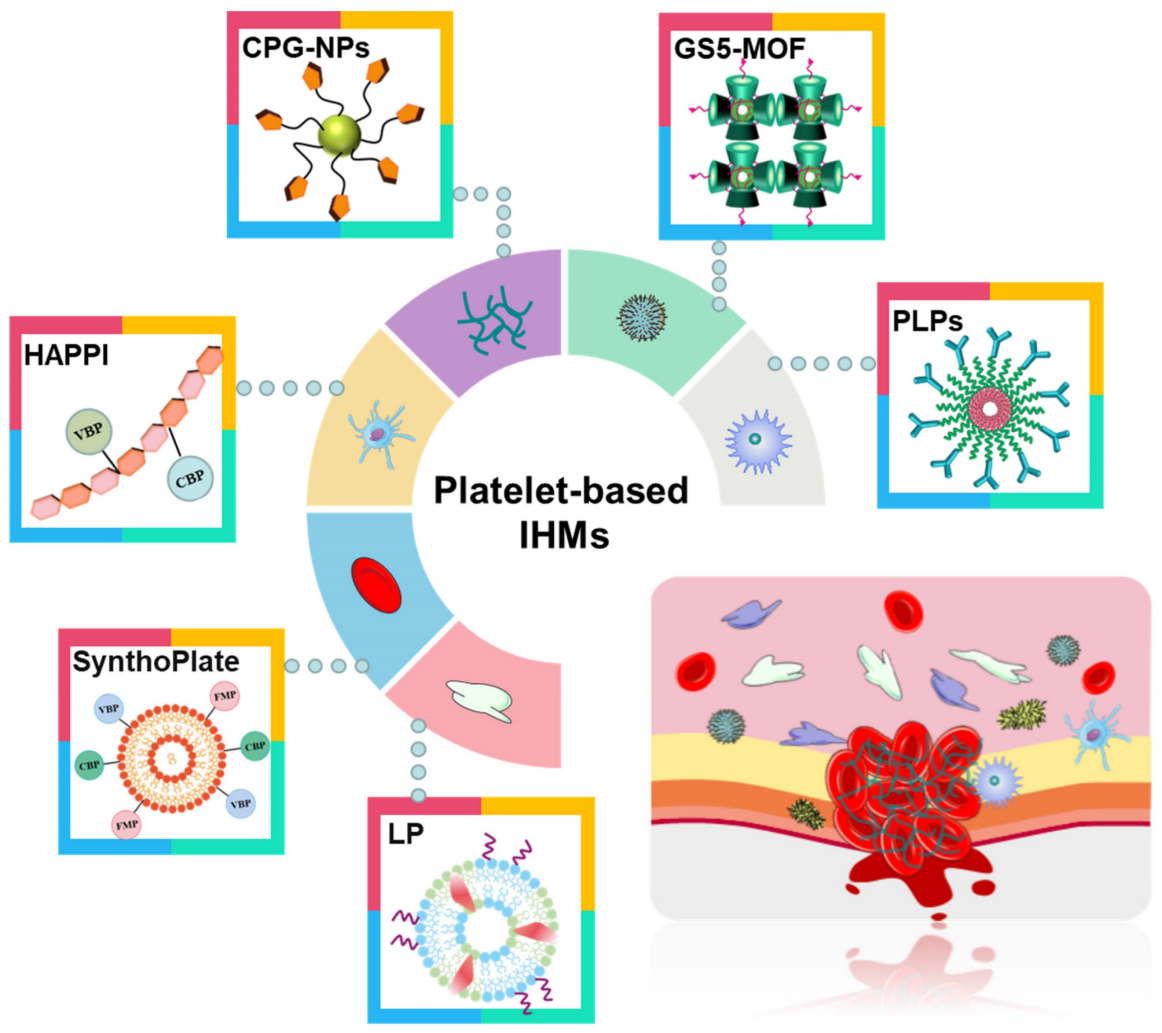
Different synthesis strategies for platelet-based IHMs. Platelets, as "first responders" in the injury process, have an innate ability to migrate to damaged blood vessel walls and gather specifically at the injury site to form platelet blockages to stop initial blood loss. Synthesis of platelet-based IHMs as an effective hemostatic pathway.

Compared with recombinant factor VIIa and tranexamic acid (TXA) 71, 72, platelet-based IHMs, also known as platelet mimicking materials or platelet substitutes, offer advantages such as low cost, easy storage, effective hemostasis, and a continuous supply. These IHMs have garnered significant interest to researchers and doctors 75, 76 due to their biochemical interaction and flexibility 73, 74. Figure 5 illustrates these associated IHMs, which can simulate, amplify, and utilize various biochemical or mechanistic platelet components 74 to target vascular injuries and stabilize bleeding 77, 78. Overall, the development of platelet-based IHMs represents a promising approach for achieving rapid hemostasis and addressing the limitations of existing interventions.

Platelet-like particles (PLPs) have been shown to bind to fibrin fibers and enhance clotting, leading to reduced bleeding time and blood loss in vivo 76. Welsch et al. 79 developed PLPs using a self-crosslinking microgel based on polyethylene glycol (PEG). Although the self-cross-linked PEG (scPEG) gel did not exhibit the same degree of network deformation ability as the ultralow cross-linked poly(N-isopropylacrylamide) (ULC pNIPAM) microgel, the fibrin-PEG-PLPs exhibited important characteristics of natural platelets. The strong adhesion between PEG-PLPs and fibrin resulted in the formation of dense fibrin areas throughout the clot. The particle-induced clotting created a synergistic effect between the collapse of local fibrin networks induced by fibrin and an increase in local particle density. The physical cross-linking between the colloidal particles and fibrin network, along with the formation of a dense fibrin area induced by particles, enhanced stability against exogenous fibrin dissolution. Thus, the strong adhesion of PEG-PLPs to newly formed fibrin clots and their non-covalent cross-linking to newly formed fibrin fibers promoted the formation of the fibrin network. Similarly, Nandi et al. 80 applied PLPs to a plasma-based fibrin network structural defect hemophilia model and investigated the effect of PLPs on fibrin structure and wound healing. They found that PLPs improved clot network density, affinity generation, and promoted fibroblast migration. This PLP technique, which maintains microgel deformation ability and platelet simulation function, is an effective strategy for controlling bleeding.

**Figure 6:**
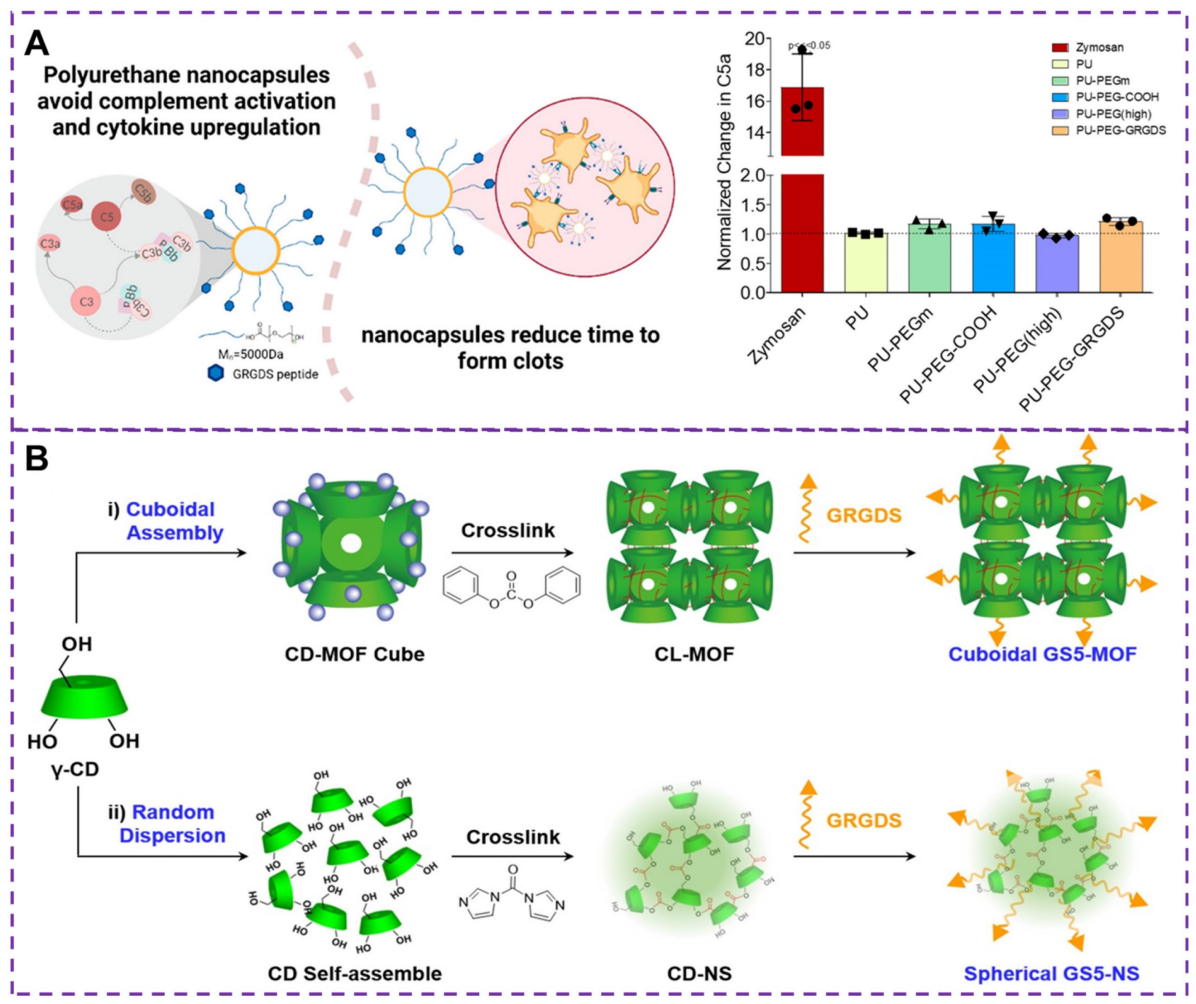
**(A)** Hemostatic materials based on polyurethane nanocapsules and the change of the protein complement C5a. The complement protein C5a is the least in non-pegylated nanocapsules and highly pegylated nanocapsules. Reproduced with permission from 86, copyright 2021, American Chemical Society. **(B)** Synthesis strategies of GS5-MOF and GS5-NS nanoparticles. GS5-MOF: The nanoparticles are synthesized by surface modification by GRGDS peptide after CD-MOF linking ester-bond crosslinking (CL-MOFs). GS5-NS: γ-cyclodextrin nanosponges (CD-NS) are modified by GRGDS. Reproduced with permission from 105, copyright 2019, Ivyspring International.

While slow infusion can reduce infusion reactions, it is not feasible for applications such as post-traumatic bleeding control. Although iron oxide formulations, infusions, and biologics 81 have certain hemostatic effect, they can complement infusion reactions, limiting the clinical use of these drugs and causing serious pathological symptoms 82, 83. Therefore, it is critical to avoiding complement activation and infusion reactions in bleeding control 84, 85. Maisha and colleagues 86 developed a new type of nanocapsule material, as shown in Figure 6A, based on polyurethane nanocapsules as a candidate material that does not activate C5a, which has been polyethylene glycolated and functionalized. Clinically relevant rotational thromboelastometry (ROTEM) tests have shown that these nanocapsules promote faster clotting and effectively maintain maximum clotting hardness, thereby promoting hemostasis. In addition, this polyurethane-based nanocapsule does not activate complement or the major pro-inflammatory cytokines, thus avoiding the induction of transfusion reactions that may cause severe inflammatory reactions, shock, or even death. Therefore, regulating the core material is a key strategy for developing safer intravenous injection of nanomedicine.

Existing nanomedicines face challenges in effectively controlling massive bleeding due to weak tissue adhesion, slow activation, and low absorption 87. While research on platelet-related vascular targeting has shown some progress 88, these nanocarriers are unable to mimic the complex biochemical interactions between natural platelets and endothelial cells, as well as lack the flexibility of platelets, hindering hemodynamic transport and the binding to damaged endothelial cells 89. To address this limitation, Wang et al. 37 developed a hybrid nanohemostat called plateletsome (LP), which harnesses the inherent hemostatic ability of platelets. Platelet exosomes, known as nanoplateletsomes, are formed by combining platelet membranes with liposomes. These platelet exosomes exhibit the advantages of liposomes and platelet membrane vesicles, enabling specific targeting of damaged blood vessels and promoting clotting similar to platelets. In a mouse model, platelet exosomes demonstrated superior hemostatic effects. Importantly, their surfaces retain the natural structure of platelet membranes, making them a unique biomimetic material. This synthesis strategy is an important way to develop efficient and safe injectable materials, which can target injured blood vessels without any drug loading and then control massive bleeding. In a similar vein, a bio-inspired "artificial PLT" called SynthoPlate has been designed 90. SynthoPlate is modified with collagen-binding peptide (CBP), fibrinogen-mimetic peptide (FMP), von Willebrand factor (vWF)-binding peptide (VBP). This modification allows for targeting of exposed procoagulant lipids, resulting in reduced blood loss and improved survival rates. Additionally, an injectable hemostat made of a synthesized polymer was reported 91, which is composed of hyaluronic acid (HA), CBP, and VBP. Hemostatic agents via polymer peptide interfusion (HAPPI) can not only selectively bind to certain protein molecules exposed in damaged blood vessels and activated platelets at the bleeding site but also promote the accumulation of activated platelets at the bleeding site, making it possible to inject from any part of the body and still reach the wound. In the mouse tail vein rupture model, the bleeding time and blood loss were reduced by 99% and 97%, respectively, while in vivo studies showed an increase in survival time by 284%. Furthermore, the freeze-dried polymer peptides can be stored stably at room temperature for several months and reconstituted during the treatment intervention process.

The electrostatic interactions between the positively charged surface of dendrimers and the negatively charged structural domains of fibrinogen enable the positively charged molecules to induce platelet aggregation 92, and this potential hemostatic effect can make them effective IHMs. Cheng et al. 93 constructed well-structured positive nanoparticles by assembling cholic acids and polyethylenimines (PEIs) through electrostatic, hydrogen bonding, and hydrophobic forces. In vitro studies showed that these assembled nanoparticles induced platelet aggregation and activation in whole blood and platelet-rich plasma. The assembled nanoparticles showed robust hemostatic effects regardless of the type of PEIs and bile acids used. Moreover, intravenously administered nanoparticles effectively reduced the number of bleeds in injured rat femoral arteries by preferentially accumulating at the injured vessel site. Therefore, PEI and bile acid-based positive nanoparticles have the potential to serve as low-cost, safe, and applicable injectable nanohaemostatic materials for managing coagulation dysfunction and controlling bleeding.

However, there are still challenges in developing effective and safe intravenous hemostatic for treating traumatic bleeding, which needs to be based on a coagulation cascade mechanism to facilitate bleeding cessation without causing excessive clotting. Currently, chitosan, a natural polysaccharide and its derivatives, have been gaining attention due to their hemostatic, antibacterial, biocompatible, and inexpensive advantages 94, 95. Chitosan-based hemostatic nanoparticles have been explored for intravenous use 96, 97. For example, Zhang et al. 98 proposed a chitosan-based nanoparticle hemostat decorated with glycine-arginine-glycine-aspartic acid-serine (GRGDS) (CPG -NPs-2000) comprising chitosan succinate (CSS) as the core and polyethylene glycol (PEG-2000) as the spacer. CPG-NPs-2000 displayed favorable drug properties, water dispersion, thermal stability, and redistribution ability. In vitro and in vivo bioassays have shown that CPG-NPs-2000 significantly accelerates coagulation and reduces bleeding time under both injured and normal conditions, and has good selectivity for platelet activation and inactivation. In a rat liver hemorrhage model, CPG-NPs-2000 showed significant hemostatic effects and safety of use parameters compared to saline, TXA and CSS. These findings suggest that CPG-NPs-2000 has a powerful potential to effectively treat severe internal bleeding, making it a valuable option for managing traumatic bleeding in both military and civilian accidents.

In addition to the strategies mentioned above for synthesizing platelet-based IHMs, researchers have also investigated their performance in extreme temperature environments and the impact of particle shape on hemostasis. While platelet-based IHMs have shown promising results in enhancing coagulation, their stability is limited in extreme temperature conditions 99. Lashof-Sullivan et al. 100 developed a hemostatic nanoparticle that can withstand higher temperatures (50 °C). These nanoparticles are composed of poly(lactic acid) (PLA), which has a high glass transition temperature, improving core stability 101. The results from liver injury experiments in rats showed that nanoparticles with a PLA core were effective in promoting hemostasis and improving survival within one hour. And these nanoparticles maintained their size, shape, and effectiveness. The presence of the RGD motif at the end of the nanoparticles allows them to bind to receptors on the surface of activated platelets, facilitating the aggregation of these activated platelets.

The surface biological function and physical mechanical properties of nanoparticle are crucial for the hemostatic process 74, 76. The shape of these synthetic particles plays a crucial role in their interaction with the blood vessel walls under flow condition 102, as well as their biological interactions with activated platelets in terms of adhesion and aggregation 103. Studies have shown that anisotropic-shaped particles exhibit enhanced adhesion in physiologically related flow environments, and the geometric shape can affect carrier's ability to evade in vivo clearance and prolong circulation time 75, 104. Therefore, controlling the shape of carriers presents an opportunity to overcome biological obstacles and improve therapeutic effects for vascular diseases. By enhancing carrier capabilities, physiological flow pattern navigation can be achieved, avoiding biological clearance, promoting adhesion to damaged vascular surfaces, and thus enhancing vascular targeting. To demonstrate the impact of the nanoparticle carrier's shape on targeted delivery of therapeutic drugs or imaging agents to injured blood vessels, He et al. 105 synthesized cubic GS5-MOFs nanoparticle, as shown in Figure 6B. The hemostatic efficiency of GS5-MOF nanoparticle in a mouse tail transection model and a rat femoral artery injury was verified through in vivo studies. The results revealed that the unique uniform size and ordered cyclodextrin framework of the GS5-MOF nanoparticles contributed to higher hemostatic efficiency and targeted delivery to injured blood vessels. These nanoparticles significantly promoted the in vitro aggregation of activated platelets through GRGDS peptide cross-linking and modification of γ-CD-MOFs surface, ultimately reducing bleeding time and blood loss in mice. Therefore, cuboidal cyclodextrin frameworks not only serve as injectable hemostatic agents for controlling non-compressible wounds but also hold enormous potential for various vascular diseases, including targeted vascular delivery, thrombosis, bleeding, and inflammation.

Consequently, to control incompressible internal hemorrhage, researchers have prepared different platelet-based IHMs that can activate the coagulation cascade and accelerate coagulation. However, it is worth noting that excessive coagulation should be avoided to prevent severe thrombosis or life-threatening symptoms. This requires effective regulation of the content of various components and the influence of the synthesis environment when designing the IHMs synthesis strategy, and careful consideration of biocompatibility, which in turn mimics the biochemical mechanism of platelets to achieve rapid hemostasis. Besides, despite their different main activities and characteristics, there is no clear distinction between various IHMs, and the development of novel IHMs based on multiple hemostatic components can be explored to overcome the limitations of single-component IHMs and enhance hemostatic performance in different applications.

### 3.2 Fibrin-based IHMs

Intravenous fibrinogen can also achieve effective hemostasis, but this strategy has some problems. For instance, the production cost of fibrinogen is high, and its isolation and reconstitution are not easily feasible 106, 107. Additionally, fibrin has a tendency to lyse, which may result in re-bleeding. To address these issues, researchers have developed fibrin-based IHMs that promote clot formation. Fibrin, derived from its precursor fibrinogen, is a natural temporary protein matrix, and the formation of an in vivo polymeric fibrin network is essential for hemostasis at the site of injury 108.

**Figure 7:**
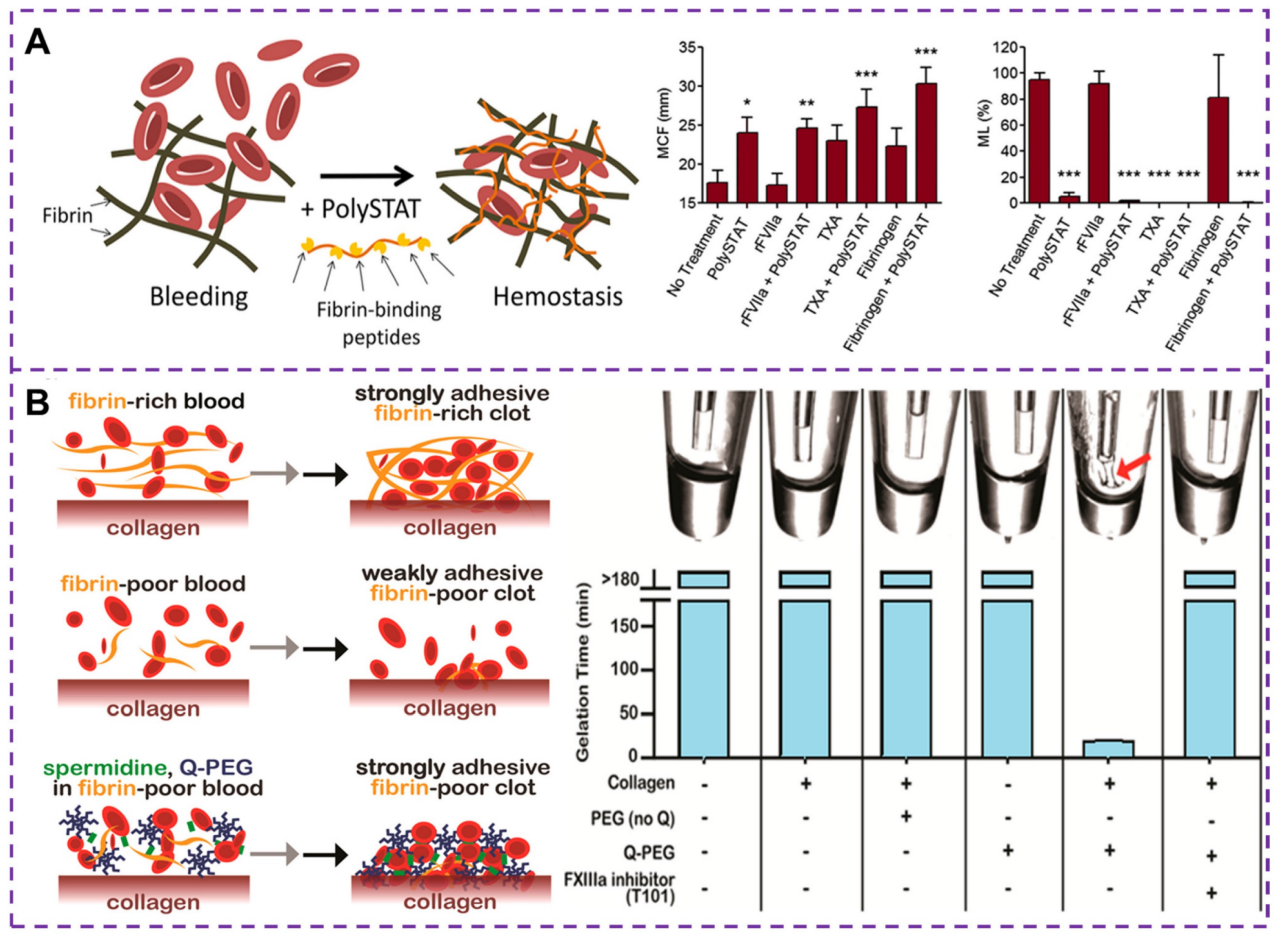
**(A)** Schematic diagram of cross-linked fibrin network formation based on PolySTAT. PolySTAT in combination with rFVIIa, TXA, and fibrinogen causes changes in the clotting process. The MCF is the maximum clot firmness, ML is the maximum lysis, and rFVIIa refers to human recombinant activated factor VII. Reproduced with permission from 112, copyright 2016, American Chemical Society. **(B)** Schematic diagram of the whole blood clot being attached to the collagen surface by FXIIIa crosslinked polymer. The Q-PEG and collagen can bind to each other under the action of FXIIIa in a gel-like phenomenon, that is, FXIIIa is responsible for binding Q-PEG to collagen through other FXIIIa substrates present in plasma, which provides the basis for improving the adhesion strength of clots. Reproduced with permission from 118, copyright 2016, American Chemical Society.

Currently, human recombinant activating factor VII 109, TXA 110 and fibrinogen concentrate 111 have been proven to promote rapid and stable fibrin formation by accelerating thrombin production, inhibiting fibrinolytic activity and replenishing depleted fibrinogen levels, respectively. These treatments effectively control bleeding and correct coagulation dysfunction in trauma patients. Fibrinogen is cleaved to form fibrin monomers, which then undergo fibrin polymerization and promote the aggregation of activated platelets by binding to GPIIb-IIIa integrin receptors in adjacent platelets. Alternatively, synthetic cross-linkers can be used to directly manipulate fibrin polymerization, improving clot formation in hemostatic situations. Chan et al. 111, 112 developed a synthetic hemostatic polymer, PolySTAT, which binds non-covalently to a variety of fibrin monomers to construct a stable cross-linked fibrin network, as depicted in Figure 7A. The results show that PolySTAT accelerates clot formation, increases the maximum stiffness of the clot, and inhibits clot lysis. Manipulating the structure of fibrin clots through physical cross-linking with synthetic polymers may provide a viable strategy for current hemostatic treatments.

Coagulation factor XIIIa (FXIIIa) is a transglutaminase that plays a crucial role in linking fibrin to collagen and fibronectin 113. This FXIIIa cross-linked polymer 114, 115, such as Q-PEG and spermidine, can be added to fibrinogen deficient plasma during the formation of coagulation networks. The presence of fibrin in a blood clot helps seal damaged blood vessels and a strongly adherent clot can improve the effectiveness of existing hemostatic agents 116. A number of adhesives have been developed for wound hemostasis, such as fibrin sealants, whose adhesion strength and hemostatic potential are closely related to fibrinogen concentration. By incorporating synthetic polymers into the fibrin clot, the mobility of the fibrin network is hindered thereby increasing its mechanical strength 117. Simultaneously, the adhesion of the clot to the blood vessel changes, and enhancing clot adhesion is also a strategy in the development of hemostatic materials. A method to increase the clot adhesion strength by adding Q-PEG polymer during the clotting process has been proposed 118, which can cross-link and copolymerize with FXIIIa, as illustrated in Figure 7B. Studies have demonstrated that the effect of Q-PEG on blood clots is mediated through the cross-linking of blood components to collagen by FXIIIa. The addition of Q-PEG to normal human plasma resulted in a non-adherent fibrinogen-deficient whole blood clot with an adhesion strength of approximately 1.9 kPa. Therefore, the synthetic polymer could increase the adhesion strength of the clot, offering a potential therapeutic approach for treating fibrinogen deficiency in cases of traumatic coagulation dysfunction.

The synergistic action of thrombin and fibrin leads to the polymerization of fibrin into a blood clot, which stops the blood flow. Apart from platelet-based IHMs, fibrin-based IHMs can also be developed for hemostatic purposes. Considering the hemostatic mechanism of fibrin, these hemostatic materials can accelerate the generation of thrombin and fibrin with the help of TXA, thereby improving clot formation and controlling bleeding. By manipulating fibrin polymerization, they can enhance the formation of clots during hemostasis, leading to the restoration of coagulation function. Fibrin-based IHMs with special structural designs can bind with substances in platelets to promote platelet hemostasis, so the rational construction of venous hemostatic agents, which take into account antimicrobial properties, biocompatibility as well as stability, is also an effective strategy.

### 3.3 Others

**Figure 8:**
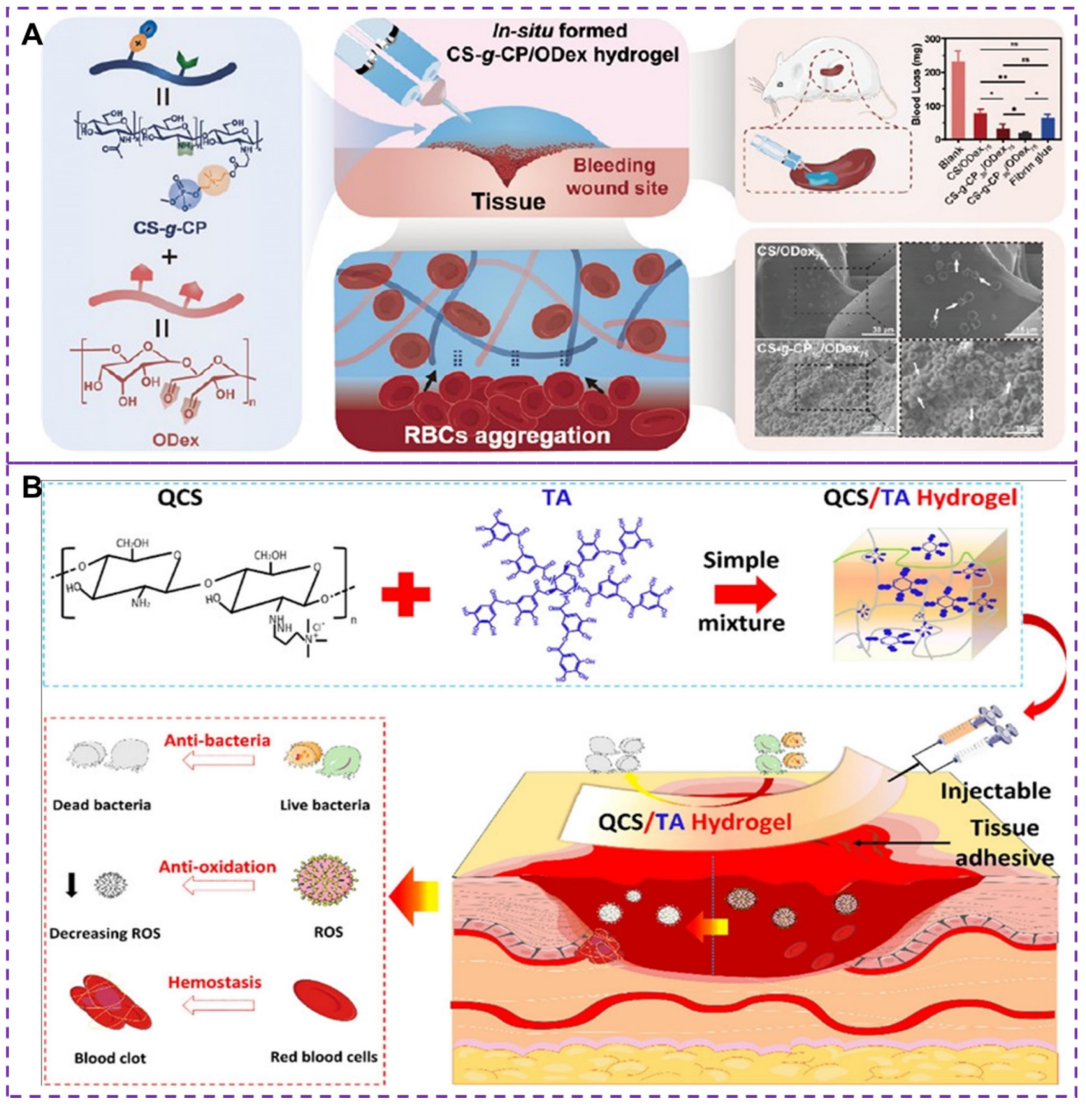
**(A)** Synthesis strategy, hemostatic mechanism and related coagulability of CS-g-CP/ODex hydrogels. The synergistic effect of CP group and chitosan enhance erythrocyte adhesion and aggregation, thus improving hemostatic performance. CS-g-CP50/ODex75 hydrogel shows higher hemostatic effect with minimal blood loss. Reproduced with permission from 120, copyright 2023, American Chemical Society. **(B)** Synthesis strategy and hemostatic mechanism of multifunctional QCS/TA hydrogels. The dynamic ionic bond and hydrogen bond crosslinking between QCS and TA make the hydrogels have good injectable, self-healing and adhesion properties. Moreover, their inherent antioxidant, antibacterial and hemostatic abilities make hemostatic materials have superior active oxygen scavenging activity, and good antibacterial ability and hemostatic ability. Reproduced with permission from 127, copyright 2022, American Chemical Society.

In addition to the methods mentioned above, such as platelet substitutes and increasing fibrin clot adhesion, there has been research exploring hemostasis through synthetic hydrogels 119, 120. However, most existing hemostatic materials still have certain limitations 121, including potential toxicity and unsatisfactory hemostatic properties. The development of injectable materials with excellent biocompatibility, self-healing and hemostatic properties is essential. Chitosan and its derivatives have low toxicity, antibacterial and adhesive properties, as well as biodegradability 122, making them suitable for construct injectable hemostatic hydrogels 34, 123, 124. The choline phosphoryl (CP) is an amphoteric structure that not only enhances biocompatibility but also plays a crucial role in stimulating the activity of red blood cells (RBCs) and improving the hemostatic properties of hemostatic hydrogels 119. Recently, Zhu et al. 120 developed an in situ injectable and self-healing hemostatic hydrogel (CS-g-CP/ODex, where CS-g-CP represents choline phosphoryl functionalized chitosan and ODex is oxidized dextran) through dynamic covalent choline phosphoryl modification of chitosan derivatives and oxidized dextran reactions. oxidized dextran). This hydrogel demonstrated enhanced erythrocyte adhesion/aggregation and coagulation, as shown in Figure 8A. The synergy between the CP group and chitosan enhanced erythrocyte adhesion and aggregation, thereby improving hemostatic performance. The scanning electron microscopy was used to evaluate the morphology of red blood cells with surface adhesion, revealing irregular deformations in shape and volume due to stronger external adhesion or physiological changes. The CS-g-CP/ODex hydrogels exhibited improved coagulation and erythrocyte adhesion/aggregation than CS/ODex hydrogels. CS-gCP50/ODex75 hydrogels demonstrated rapid gelation time, satisfactory rupture pressure, good biocompatibility, adhesion properties and mechanical properties. However, it should be noted that although the injectable hydrogels can help promote blood clotting and stop bleeding, there is a potential concern regarding the formation of blood clots after bleeding ceases. This can have serious implications if a clot blocks a blood vessel, impeding blood flow.

Tannic acid (TA) is a natural polyphenol with antibacterial, biodegradable, and antioxidant properties 125. The quaternary ammonium chitosan (QCS), as an antibacterial cationic polymer, is an effective raw material for the development of durable antibacterial hemostatic materials 126. Recently, a multifunctional QCS/TA hydrogel has been developed as a novel hemostatic material 127, exhibiting excellent injectability, adhesion strength, self-healing ability, antibacterial properties, and hemostatic capacities, as depicted in Figure 8B. The hydrogel achieves its properties through the rapid and reversible formation of ionic and hydrogen bonds between QCS and TA, allowing it to seamlessly integrate into irregularly shaped wounds through shear dilution injection or in situ formation. The experimental results have demonstrated that the QCS/TA2.5 hydrogel exhibits good adhesion, biocompatibility, rapid hemostatic effect on arterial and deep incompressible wounds. Moreover, the hydrogel effectively stops bleeding in such wounds and demonstrates strong antimicrobial activity, reducing bacterial infections in full skin wounds. Nevertheless, it is important to note that some hydrogels, particularly those containing synthetic or modified materials, may carry a higher risk of inducing thrombus formation compared to others. The risk of thrombus formation depends on several factors, including the composition and properties of the hydrogel, the location and method of injection, and the individual susceptibility of the patient to clotting. To mitigate the adverse effects of thrombosis, several measures can be taken, such as optimizing the material composition and properties of hydrogels, as well as conducting in vitro and in vivo studies to assess the safety and efficacy. Therefore, it is critical to address the potential risks of thrombosis associated with injectable hydrogels in terms of tissue healing and health recovery.

Regardless of the synthesis strategy, these IHMs are designed to interact with the blood and the surrounding tissues to facilitate stable clot formation. The structures and compositions of IHMs play a crucial role in their effectiveness. The materials should have suitable surface properties to promote platelet adhesion and activation as well as to facilitate coagulation factor interactions. Additionally, the materials must be biocompatible, non-toxic, and able to be biodegraded or cleared from the body over time. It's important to note that the selection of IHMs depends on the specific clinical situation and the severity of bleeding.

## 4. Performance requirements of IHMs

**Figure 9:**
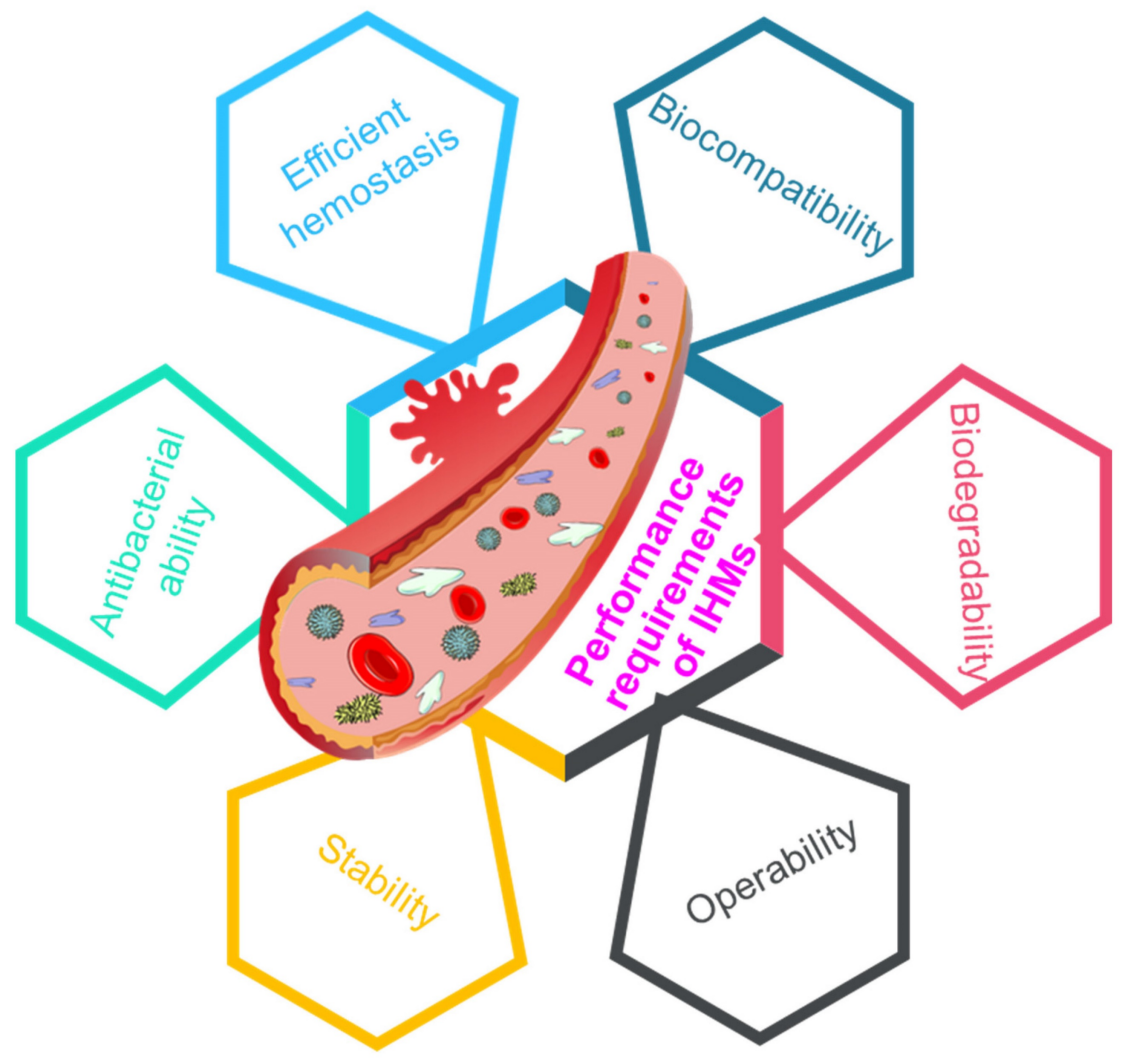
Performance requirements of IHMs, including efficient hemostasis, biocompatibility, biodegradability, operability, stability, and antibacterial ability, which can affect the clinical applications of IHMs.

Multifunctional materials bring changes to people's lives and promote social progress 128-130, and the requirements for the performance of hemostatic material are increasing. To promote rapid wound hemostasis and healing, it is an inevitable trend to develop IHMs high efficiency, biocompatibility, degradability, workability, stability and antibacterial properties 32, 131, as depicted in Figure 9.

### 4.1 Efficient hemostasis

Excellent adhesion and hemostatic functionality enable hemostatic materials to immediately repair acute tissue damage 132. The utilization of IHMs in first aid can significantly enhance hemostatic efficiency. Since bleeding can occur in any part of the body, IHMs must cater to the diverse needs of different regions. Recently developed platelet-like structures, such as LP 37 and SynthoPlate 90 materials, have demonstrated cell aggregation, adhesion, and coagulation promotion properties, effectively stimulating clot formation and facilitating its secure fixation at the wound site to achieve rapid hemostasis. The good hemostatic effect is the first and most critical performance requirement for the design and synthesis of IHMs.

### 4.2 Biocompatibility

Good biocompatibility ensures that hemostatic materials can rapidly control bleeding and be easily applied to different types of wounds 34, 133. Since IHMs are directly into the human body, they must exhibit excellent biocompatibility and safety during use, exemplified by materials like PEG-PLP. Specifically, they should have non-toxic, non-allergenic, non-allergic and other safety performance requirements. The formation of clotting factors, such as platelets, at the wound site represents a pivotal step in hemostasis, but excessive hemostasis results in the formation of clots in non-ruptured vessels. To prevent complications such as thrombosis following excessive hemostasis, it is crucial for hemostatic materials to degrade within the shortest feasible timeframe by regulating the content of various components in the blood.

### 4.3 Biodegradability

Biodegradable hemostatic materials, characterized by simplicity in preparation, cost-effectiveness, portability, and ease of use, hold significant potential as novel materials for rapid hemostasis and wound healing promotion 134, 135. These IHMs need to degrade and be absorbed within a suitable timeframe without adversely affecting normal physiological functions. Consequently, performance requirements for these material include degradation time, stability of the degradation products 112, and the capacity for safe absorption and metabolism within the human body.

### 4.4 Operability

The convenience and ease of handling during hemostasis are important properties of IHMs 136. A high degree of operability in IHMs can greatly improve hemostasis, patient outcomes, as well as reduce the risk and cost of subsequent procedures. The HAPPI materials have the ability to bind to protein molecules and platelets, making them to be injected from any part of the body and thus offering a high degree of operability. The operability of IHMs encompasses several aspects, including the form of preparation, injection method, syringe design, and injection dosage. To achieve operability of IHMs, the following aspects need to be considered: firstly, the form of preparation should be simple and user-friendly, tailored to the type of trauma and requirements; secondly, the choice of injection method should consider the specific bleeding site and should be easy to implement. Thirdly, syringes should be ergonomically designed to facilitate ease of use for the operator while ensuring good protection. Fourthly, the precise control of the injection volume is one of the key items in the use of IHMs. Inadequate or excessive doses can lead to unsatisfactory hemostasis. Therefore, injections should strictly follow the prescribed dosage to maintain the accuracy and stability of the injected dose.

### 4.5 Stability

Stability refers to the ability of IHMs to maintain their performance, quality, and activity during storage and transportation over long periods of time. The long-term performance stability ensures that IHMs can be used with the desired therapeutic effect. Platelets have a relatively short shelf life 60, 137, and some of the IHMs developed even need to be stored in a cryogenic environment. A longer shelf life means a more reliable material with a wider range of uses, as long as its performance is guaranteed. This stability is influenced by a combination of factors, including physical, chemical, and biological factors. The physical factors include temperature and humidity; the chemical factors include light, oxidation, and hydrolysis; while the biological factors refer to the biochemical reactions that occur within the body of the material. In actual use, these factors must be controlled during the storage and transportation of IHMs in order to guarantee their stability and reliability, thus better safeguarding the hemostatic effect of patients. For example, a PLA-based nanoparticle 100 has been developed that guarantees hemostasis with good stability.

### 4.6 Antibacterial ability

Antibacterial ability 138, 139 refers to the ability of a material to inhibit or kill bacteria, which can be influenced by its inherent properties or the presence of added antimicrobial agents. The invasion of living tissue by microorganisms can lead to bacterial infections, hindering the wound healing process and causing severe damage to blood vessels and cells 140, 141. Additionally, some IHMs are prone to bacteria contamination, especially those that need to be stored for a long time or even reused. Therefore, it is beneficial to create IHMs with antimicrobial activity to counteract microbial interference. There are various approaches to enhance the antibacterial ability of hemostasis materials for injection, such as incorporating antimicrobial components to prevent bacterial growth or selecting materials with stronger inherent antibacterial properties for manufacturing IHMs. It is crucial to maintain a sterile environment during the production of IHMs to ensure freedom from bacterial contamination. The development of effective antimicrobial materials for drug-resistant bacteria is a key focus of future research in the field of hemostatic materials. The PLPs combined with antimicrobial metals, such as nanosilver-containing PLPs, have demonstrated the ability to retain their original hemostatic function while exhibiting good antibacterial abilities 142, 143. The formation of spherical silver nanoparticles has been found to inhibit bacterial growth, but their poor biosafety due to the use of organic or inorganic reagents during their preparation has limited their widespread adoption 144, 145. The search for materials with both hemostatic and antibacterial properties is an important avenue of research for the development of rapid hemostatic materials.

The IHMs are a new type of rapid hemostatic material that has been developed in recent years. The demand for high-performance IHMs continues to rise due to the need to combat terrorism. Therefore, it is important to further improve the hemostasis rate of IHMs while ensuring their good biosafety, workability, stability, and antibacterial properties, ultimately promoting their clinical application.

## 5. Conclusion and perspective

Over the past few decades, we have witnessed rapid advancements in hemostatic materials. As an emerging class of IHMs, these materials offer a promising solution for emergency situations involving massive bleeding. Due to their excellent hemostatic properties, they have garnered significant attention from scientific researchers and medical professionals. Extensive studies have been conducted on their hemostatic mechanism, coagulation characteristics, and synthesis strategies, which have been applied to various models such as hemophilia models with structural defects in the fibrin network, mouse models with torn tail veins, and rat models with tail amputations or liver/spleen injuries. These studies are very important to reveal the bleeding process and hemostatic mechanism as well as the practical application of hemostasis. Based on this, this paper firstly analyzes the physiological hemostatic process and related mechanisms, then discusses the synthesis strategies of different IHMs, comprehensively combs the performance requirements of IHMs, and finally prospects the future development and challenges of IHMs. This review serves as a comprehensive summary of IHMs and establishes a foundation for the design, optimization, and application of novel hemostatic materials.

With the development of science and technology and medical progress, more requirements are put forward for the performance of IHMs. The ideal IHMs should possess excellent antibacterial and hemostatic capabilities, no damage to human skin, easy to process, economical, and portable to meet the needs of medical applications. Although currently available injectable biological hemostatic materials have shown significant advantages and potential in controlling internal bleeding, they still have certain limitations, such as varying application scopes. Therefore, continuous improvement and the synthesis of composite materials are necessary to enhance their performance. Based on the finding of this review, the future development directions of IHMs can be summarized as follows:

### (1) Design of multifunctional structure

The wound healing process is complex, and current treatments with most hemostatic materials primarily focus on skin repair, leaving a scarcity of materials that can effectively treat internal bleeding. This is an area that deserves significant attention. With advancements in science and technology, hemostasis is no longer the sole ultimate goal. Hemostasis is an important first step in the wound healing process. Hemostatic materials with multifunctional structure designs offer significant advantages in practical applications. The use of various materials with hemostatic activity helps overcome the inherent defects of single hemostatic components and enhances hemostatic performance in different application scenarios. Furthermore, IHMs can be combined with other functional materials such as pain relief, anti-infection, wound healing, and the promotion of epidermal cell growth, fully utilizing the functions of various hemostatic materials beyond hemostasis. Through modification or composite technology, multifunctional composite hemostatic materials can be developed to further enhance the efficacy of injectable trauma first aid hemostatic materials. Additionally, the design of IHMs is becoming increasingly intelligent, with a greater focus on to post-hemostasis management. Endowing hemostatic materials with various biomedical functions is of great significance, including promoting wound healing and achieving visual monitoring of bleeding locations. Functionalized nanoscale hemostatic materials with diagnostic and therapeutic capabilities offer more advantages than conventional hemostatic materials, and their development will greatly facilitate their practical application in various bleeding control and wound healing scenarios. Therefore, the development of hemostatic materials with multifunctional structures and the improvement of hemostatic performance and treatment effects are likely to be the future trends in medical IHMs.

### (2) Optimization of synthesis strategy

Hemostatic materials based on platelet and fibrin structures can be designed to actively participate in or promote clot contraction, leading to a stronger clot formation and more effective cessation of bleeding. This offers promising opportunities for the development of hemostatic materials. However, the synthesis of IHMs is still in a relatively early stage of development, with many synthesis strategies not yet matured. Additionally, most of the existing strategies are complex and time-consuming. Therefore, the rational optimization of current synthesis strategies to prepare new hemostatic materials and enhance their hemostatic performance have become the main research hotspot. Developing a simple and low-cost synthesis strategy remains a huge challenge. Faster and simpler chemical reactions would facilitate high-yield material production at a reasonable cost and promote practical clinical applications. Furthermore, novel synthesis methods should take into account the principles of green chemistry and the cost of components. By adjusting the composition of hemostatic materials and utilizing synthetic techniques such as surface modification, these materials can possess multifunctionality. IHMs can also be synthesized in combination with other chemicals to increase binding to the clotting interface and improve hemostasis and clot formation. In addition, the synthesis process should consider the physical and chemical properties of each component of the hemostatic material to promote their synergistic effects. Considering the limitations of existing synthesis methods, there is still much more to discover regarding the full potential of novel IHMs.

### (3) Improvement of hemostatic mechanism

The different hemostatic mechanisms and effects of IHMs pose a challenge in fully utilizing their characteristics in clinical treatment and efficiently leveraging their advantages. To achieve a more satisfactory hemostatic effect, a variety of hemostatic materials are used based on their different properties, allowing for the optimal utilization of their advantages. Therefore, it is crucial to understand how to change the blood components and the coagulation cascade during hemostasis to effectively enhance their maximum effect. This necessitates a greater focus on the hemostatic process and mechanism, delving deeper into the internal function theory, such as exploring the cellular and molecular-level hemostatic mechanisms within the body. Platelet-based IHMs not only participate in and regulate the physiological hemostasis process, but also need to consider the infusion-related complement response, which increases the complexity to the hemostasis process. Consequently, a systematic and in-depth study of the hemostatic mechanism is necessary. Additionally, the development of new hemostatic materials and a thorough investigation of their hemostatic mechanism are crucial aspects of the next generation of nano IHMs, providing a mechanism guarantee for future clinical applications.

### (4) Establishment of systematic evaluation system

The comprehensively evaluate the effectiveness of injectable nanoscale hemostatic materials in assessing the macroscopic system of materials remains challenging. This limitation greatly hampers the application of such materials in hemostasis. Therefore, a comprehensive and systematic evaluation of the biocompatibility and hemostatic performance of hemostatic materials is essential. Currently, research on IHMs is still in its initial stages and lacks a standardized evaluation system. By optimizing the evaluation methods used to assess the effectiveness of IHMs, we can provide a reference for their further development and preclinical research. With the rapid emergence of new hemostatic materials, it becomes imperative to establish an objective and effective evaluation system to comprehensively assess their hemostatic effects. A combination of in vitro and in vivo evaluation methods can be employed to qualitatively and quantitatively evaluate the hemostatic effect of materials. In vitro evaluation offers a simple, fast, and direct method to assess clotting formation, platelet adhesion and activation, fibrinogen adsorption, clotting efficiency, thrombin generation, and hemostatic efficacy through a series of tests. On the other hand, in vivo evaluation, although more costly and complex, primarily focuses on indicators such as blood loss, healing rates, and survival rates. A comprehensive evaluation system that effectively combines both evaluation methods is the foundation for promoting and improving the hemostatic performance of hemostatic materials.

### (5) Promotion of clinical application

Although numerous IHMs have been developed, their clinical translation remains inadequate. Most of these hemostatic materials are tested in animal models such as mice, rats, pigs, and rabbits, which differ significantly from humans in terms of physiology and hemodynamics. The lack of sufficient statistical data on hemostatic materials in actual clinical practice makes it challenging to conduct clinical trials, thus hindering the development of IHMs. Safety is a fundamental and critical concern for the practical application of IHMs. The use of IHMs must ensure the biocompatibility of their degradation products and minimize the risk of inflammatory reactions in vivo. The degradation rate of IHMs should align with tissue repair process. The complex synthesis of IHMs poses difficulties in ensuring the stability and applicability, which remains an unresolved issue. Moreover, hemostatic materials are usually delivered directly to the bleeding site through a syringe. Changing the traditional hemostatic dosage form or optimizing the hemostatic administration method will greatly improve the viability in emergency situations. Addressing these challenges and issues requires collaboration between laboratory researchers and clinicians. Future studies should focus on the long-term reactions and potential risks associated with the degradation products of IHMs.

In summary, most of the current technologies for the synthesis of IHMs are still in the early stages of development. It is crucial to synthesize novel hemostatic materials that are fast, efficient, safe, and portable. Injectable trauma first aid hemostatic materials hold great promise in saving lives during tactical combat injuries and represent a key research and development area in medical biomaterials. With advancements in science and technology and further research conducted both domestically and internationally, the new generation of IHMs will definitely be put into application to meet the needs of patients for rapid hemostasis and rehabilitation, which will also accelerate the translation of hemostatic materials from basic research to clinical application, ultimately ensuring the preservation of human lives.

## Abbreviations

ADP: adenosine diphosphate
CBP: collagen-binding peptide
CD-MOFs: cuboidal and biocompatible γ-cyclodextrin metal-organic frameworks
CD-NS: cuboidal and biocompatible γ-cyclodextrin nanosponges
CP: choline phosphoryl
CSS: chitosan succinate
FMP: fibrinogen-mimetic peptide
GRGDS: glycine-arginine-glycine-aspartic acid-serine
HA: hyaluronic acid
HAPPI: hemostatic agents via polymer peptide interfusion
IHMs: injectable hemostatic materials
LP: plateletsome
MCF: maximum clot firmness
ML: maximum lysis
ODex: oxidized dextran
PEG: polyethylene glycol
PEIs: polyethylenimines
PLA: poly(lactic acid)
PLPs: Platelet-like particles
QCS: quaternary ammonium chitosan
RBCs: red blood cells
rFVIIa: human recombinant activated factor VII
ROTEM: rotational thromboelastometry
scPEG: self-cross-linked polyethylene glycol
TA: Tannic acid
TXA: tranexamic acid
ULC pNIPAM: ultralow cross-linked poly(N-isopropylacrylamide
VBP: von willebrand factor-binding peptide
vWF: von willebrand factor

## References

[1] Rodrigues M, Kosaric N, Bonham CA, Gurtner GC (2019). Wound Healing: A Cellular Perspective. Physiol Rev.

[2] Zhang X, Yao D, Zhao W, Zhang R, Yu B, Ma G (2021). Engineering Platelet-Rich Plasma Based Dual-Network Hydrogel as a Bioactive Wound Dressing with Potential Clinical Translational Value. Adv Funct Mater.

[3] Luo M, Shaitan K, Qu X, Bonartsev AP, Lei B (2022). Bioactive rare earth-based inorganic-organic hybrid biomaterials for wound healing and repair. Appl Mater Today.

[4] Homaeigohar S, Boccaccini AR (2020). Antibacterial biohybrid nanofibers for wound dressings. Acta Biomater.

[5] Fahimirad S, Ajalloueian F (2019). Naturally-derived electrospun wound dressings for target delivery of bio-active agents. Int J Pharm.

[6] Opneja A, Kapoor S, Stavrou EX (2019). Contribution of platelets, the coagulation and fibrinolytic systems to cutaneous wound healing. Thromb Res.

[7] Fan C, Xu Q, Hao R, Wang C, Que Y, Chen Y (2022). Multi-functional wound dressings based on silicate bioactive materials. Biomaterials.

[8] Hong Y, Zhou F, Hua Y, Zhang X, Ni C, Pan D (2019). A strongly adhesive hemostatic hydrogel for the repair of arterial and heart bleeds. Nat Commun.

[9] Yuan H, Chen L, Hong FF (2020). A Biodegradable Antibacterial Nanocomposite Based on Oxidized Bacterial Nanocellulose for Rapid Hemostasis and Wound Healing. ACS Appl Mater Interfaces.

[10] Qin X-H, Labuda K, Chen J, Hruschka V, Khadem A, Liska R (2015). Development of Synthetic Platelet-Activating Hydrogel Matrices to Induce Local Hemostasis. Adv Funct Mater.

[11] Hamada SR, Rosa A, Gauss T, Desclefs J-P, Raux M, Harrois A (2018). Development and validation of a pre-hospital “Red Flag” alert for activation of intra-hospital haemorrhage control response in blunt trauma. Critical Care.

[12] Spinella PC (2017). Zero preventable deaths after traumatic injury: An achievable goal. J Trauma Acute Care.

[13] Hickman DA, Pawlowski CL, Sekhon UDS, Marks J, Gupta AS (2018). Biomaterials and Advanced Technologies for Hemostatic Management of Bleeding. Adv Mater.

[14] Zia J, Kimball J, Rolfes C, Hahn J-O, Inan OT (2020). Enabling the assessment of trauma-induced hemorrhage via smart wearable systems. Sci Adv.

[15] King DR (2019). Initial Care of the Severely Injured Patient. N Engl J Med.

[16] Montazerian H, Davoodi E, Baidya A, Baghdasarian S, Sarikhani E, Meyer CE (2022). Engineered Hemostatic Biomaterials for Sealing Wounds. Chem Rev.

[17] Cui C, Liu W (2021). Recent advances in wet adhesives: Adhesion mechanism, design principle and applications. Prog Polym Sci.

[18] Hickman DA, Pawlowski CL, Shevitz A, Luc NF, Kim A, Girish A (2018). Intravenous synthetic platelet (SynthoPlate) nanoconstructs reduce bleeding and improve 'golden hour' survival in a porcine model of traumatic arterial hemorrhage. Sci Rep.

[19] Zou C-Y, Li Q-J, Hu J-J, Song Y-T, Zhang Q-Y, Nie R (2022). Design of biopolymer-based hemostatic material: Starting from molecular structures and forms. Materials Today Bio.

[20] Morgan CE, Dombrowski AW, Rubert Pérez CM, Bahnson ESM, Tsihlis ND, Jiang W (2016). Tissue-Factor Targeted Peptide Amphiphile Nanofibers as an Injectable Therapy To Control Hemorrhage. ACS Nano.

[21] Dowling MB, Smith W, Balogh P, Duggan MJ, MacIntire IC, Harris E (2015). Hydrophobically-modified chitosan foam: description and hemostatic efficacy. J Surg Res.

[22] Peng HT (2020). Hemostatic agents for prehospital hemorrhage control: a narrative review. Military Med Res.

[23] Lashof-Sullivan M, Shoffstall A, Lavik E (2013). Intravenous hemostats: challenges in translation to patients. Nanoscale.

[24] Zhao X, Liang Y, Guo B, Yin Z, Zhu D, Han Y (2021). Injectable dry cryogels with excellent blood-sucking expansion and blood clotting to cease hemorrhage for lethal deep-wounds, coagulopathy and tissue regeneration. Chem Eng J.

[25] Butenas S, Mann KG, Butenas (2002). Blood Coagulation. Biochemistry (Moscow).

[26] Malik A, Rehman FU, Shah KU, Naz SS, Qaisar S (2021). Hemostatic strategies for uncontrolled bleeding: A comprehensive update. J Biomed Mater Res B.

[27] Pourshahrestani S, Zeimaran E, Djordjevic I, Kadri NA, Towler MR (2016). Inorganic hemostats: The state-of-the-art and recent advances. Mat Sci Eng C.

[28] Sung YK, Lee DR, Chung DJ (2021). Advances in the development of hemostatic biomaterials for medical application. Biomater Res.

[29] Versteeg HH, Heemskerk JWM, Levi M, Reitsma PH (2013). New Fundamentals in Hemostasis. Physiol Rev.

[30] Pathak A, Zhao R, Monroe DM, Roberts HR, Sheridan BC, Selzman CH (2006). Thrombin generation in vascular tissue. J Thromb Haemost.

[31] Terent'eva VA, Sveshnikova AN, Panteleev MA (2017). Biophysical mechanisms of contact activation of blood-plasma clotting. Biophysics.

[32] Behrens AM, Sikorski MJ, Kofinas P (2014). Hemostatic strategies for traumatic and surgical bleeding. J Biomed Mater Res A.

[33] Wang L, You X, Dai C, Tong T, Wu J (2020). Hemostatic nanotechnologies for external and internal hemorrhage management. Biomater Sci.

[34] Guo B, Dong R, Liang Y, Li M (2021). Haemostatic materials for wound healing applications. Nat Rev Chem.

[35] Pourshahrestani S, Zeimaran E, Kadri NA, Mutlu N, Boccaccini AR (2020). Polymeric Hydrogel Systems as Emerging Biomaterial Platforms to Enable Hemostasis and Wound Healing. Adv Healthc Mater.

[36] Sang Y, Roest M, de Laat B, de Groot PG, Huskens D (2021). Interplay between platelets and coagulation. Blood Rev.

[37] Wang H, Zhu Y, Zhang L, Liu H, Liu C, Zhang B (2022). Nanoplateletsomes for rapid hemostasis performance. Chin Chem Lett.

[38] Xiao L, Ma Y, Crawford R, Mendhi J, Zhang Y, Lu H (2022). The interplay between hemostasis and immune response in biomaterial development for osteogenesis. Mater Today.

[39] Kerris EWJ, Hoptay C, Calderon T, Freishtat RJ (2020). Platelets and Platelet Extracellular Vesicles in Hemostasis and Sepsis. J Investig Med.

[40] Alamin AA (2020). The Role of Red Blood Cells in Hemostasis. Semin Thromb Hemost.

[41] Noubouossie DF, Henderson MW, Mooberry M, Ilich A, Ellsworth P, Piegore M (2020). Red blood cell microvesicles activate the contact system, leading to factor IX activation via 2 independent pathways. Blood.

[42] Weisel JW, Litvinov RI (2019). Red blood cells: the forgotten player in hemostasis and thrombosis. J Thromb Haemost.

[43] Li G, Quan K, Xu C, Deng B, Wang X (2018). Synergy in thrombin-graphene sponge for improved hemostatic efficacy and facile utilization. Colloid Surf B.

[44] Smeets R, Gerhards F, Stein J, Pereira Paz RM, Vogt S, Pautke C (2011). A novel hemostatic delivery device for thrombin: Biodegradable poly(D,L-lactide-co-glycolide) 50:50 microspheres. J Biomed Mater Rest A.

[45] Beudert M, Gutmann M, Lühmann T, Meinel L (2022). Fibrin Sealants: Challenges and Solutions. ACS Biomater Sci Eng.

[46] Lundin JG, McGann CL, Daniels GC, Streifel BC, Wynne JH (2017). Hemostatic kaolin-polyurethane foam composites for multifunctional wound dressing applications. Mater Sci Eng C.

[47] Li J, Han J, Sun Q, Wang Y, Mu Y, Zhang K (2018). Biosynthetic calcium-doped biosilica with multiple hemostatic properties for hemorrhage control. J Mater Chem B.

[48] Chen J, Qiu L, Li Q, Ai J, Liu H, Chen Q (2021). Rapid hemostasis accompanied by antibacterial action of calcium crosslinking tannic acid-coated mesoporous silica/silver Janus nanoparticles. Mater Sci Eng C.

[49] Briggs A, Askari R (2016). Damage control resuscitation. Int J Surg.

[50] Pohlman TH, Walsh M, Aversa J, Hutchison EM, Olsen KP, Lawrence Reed R (2015). Damage control resuscitation. Blood Rev.

[51] Wang H, Yang L (2023). Dielectric constant, dielectric loss, conductivity, capacitance and model analysis of electronic electroactive polymers. Polym Test.

[52] Wang H, Yang L, Tian A, Huang B (2023). Fractal recognition and contact characteristics of ionic electroactive polymer interface based on microstructure analysis. Tribol Int.

[53] Yang L, Wang H, Zhang F, Yang Y, Qu S, Leng D (2023). Synthetic technologies, property enhancements and versatile applications of calcium copper titanate: A review. Nano Energy.

[54] Wang H, Yang L, Yang Y, Zhang D, Tian A (2023). Highly flexible, large-deformation ionic polymer metal composites for artificial muscles: Fabrication, properties, applications, and prospects. Chem Eng J.

[55] Yang L, Wang H, Fang S, Li M (2023). Research progress on energy storage performance enhancement strategies for polyvinylidene fluoride-based composites. J Alloys Compd.

[56] Terentes-Printzios D, Ioakeimidis N, Rokkas K, Vlachopoulos C (2022). Interactions between erectile dysfunction, cardiovascular disease and cardiovascular drugs. Nat Rev Cardiol.

[57] Ding K, Zheng C, Sun L, Liu X, Yin Y, Wang L (2020). NIR light-induced tumor phototherapy using ICG delivery system based on platelet-membrane-camouflaged hollow bismuth selenide nanoparticles. Chin Chem Lett.

[58] Yeaman MR (2010). Platelets in defense against bacterial pathogens. Cell Mol Life Sci.

[59] Olsson M, Bruhns P, Frazier WA, Ravetch JV, Oldenborg P-A (2005). Platelet homeostasis is regulated by platelet expression of CD47 under normal conditions and in passive immune thrombocytopenia. Blood.

[60] Bynum JA, Meledeo MA, Peltier GC, McIntosh CS, Taylor AS, Montgomery RK (2019). Evaluation of a lyophilized platelet-derived hemostatic product. Transfusion.

[61] Shukla M, Sekhon UDS, Betapudi V, Li W, Hickman DA, Pawlowski CL (2017). In vitro characterization of SynthoPlate™ (synthetic platelet) technology and its in vivo evaluation in severely thrombocytopenic mice. J Thromb Haemost.

[62] Stanworth SJ, Navarrete C, Estcourt L, Marsh J (2015). Platelet refractoriness - practical approaches and ongoing dilemmas in patient management. Br J Haematol.

[63] Prodger CF, Rampotas A, Estcourt LJ, Stanworth SJ, Murphy MF (2020). Platelet transfusion: Alloimmunization and refractoriness. Semin Hematol.

[64] Pidcoke HF, McFaul SJ, Ramasubramanian AK, Parida BK, Mora AG, Fedyk CG (2013). Primary hemostatic capacity of whole blood: a comprehensive analysis of pathogen reduction and refrigeration effects over time. Transfusion.

[65] Slichter SJ, Corson J, Jones MK, Christoffel T, Pellham E, Bailey SL (2014). Exploratory studies of extended storage of apheresis platelets in a platelet additive solution (PAS). Blood.

[66] Fitzpatrick GM, Cliff R, Tandon N (2013). Thrombosomes: a platelet-derived hemostatic agent for control of noncompressible hemorrhage. Transfusion.

[67] Burdette AJ, Andrew Pratt G, Campagna MV, Sheppard FR (2017). Evaluation of a new generation platelet-derived hemostatic agent in a rabbit thrombocytopenic model. Thromb Res.

[68] Jung H, Kang YY, Mok H (2019). Platelet-derived nanovesicles for hemostasis without release of pro-inflammatory cytokines. Biomater Sci.

[69] Lambert MP, Sullivan SK, Fuentes R, French DL, Poncz M (2013). Challenges and promises for the development of donor-independent platelet transfusions. Blood.

[70] Thon JN, Mazutis L, Wu S, Sylman JL, Ehrlicher A, Machlus KR (2014). Platelet bioreactor-on-a-chip. Blood.

[71] Pusateri AE, Weiskopf RB, Bebarta V, Butler F, Cestero RF, Chaudry IH (2013). Tranexamic Acid and Trauma: Current Status and Knowledge Gaps With Recommended Research Priorities. Shock.

[72] Boffard KD, Riou B, Warren B, Choong PIT, Rizoli S, Rossaint R (2005). Recombinant Factor VIIa as Adjunctive Therapy for Bleeding Control in Severely Injured Trauma Patients: Two Parallel Randomized, Placebo-Controlled, Double-Blind Clinical Trials. J Trauma Acute Care.

[73] Nandi S, Brown AC (2016). Platelet-mimetic strategies for modulating the wound environment and inflammatory responses. Exp Biol Med.

[74] Anselmo AC, Modery-Pawlowski CL, Menegatti S, Kumar S, Vogus DR, Tian LL (2014). Platelet-like Nanoparticles: Mimicking Shape, Flexibility, and Surface Biology of Platelets To Target Vascular Injuries. ACS Nano.

[75] Modery-Pawlowski CL, Tian LL, Pan V, McCrae KR, Mitragotri S, Sen Gupta A (2013). Approaches to synthetic platelet analogs. Biomaterials.

[76] Brown AC, Stabenfeldt SE, Ahn B, Hannan RT, Dhada KS, Herman ES (2014). Ultrasoft microgels displaying emergent platelet-like behaviours. Nat Mater.

[77] Long M, Zhang Y, Huang P, Chang S, Hu Y, Yang Q (2018). Emerging Nanoclay Composite for Effective Hemostasis. Adv Funct Mater.

[78] Hansen CE, Myers DR, Baldwin WH, Sakurai Y, Meeks SL, Lyon LA (2017). Platelet-Microcapsule Hybrids Leverage Contractile Force for Targeted Delivery of Hemostatic Agents. ACS Nano.

[79] Welsch N, Brown AC, Barker TH, Lyon LA (2018). Enhancing clot properties through fibrin-specific self-cross-linked PEG side-chain microgels. Colloid Surf B.

[80] Nandi S, Sommerville L, Nellenbach K, Mihalko E, Erb M, Freytes DO (2020). Platelet-like particles improve fibrin network properties in a hemophilic model of provisional matrix structural defects. J Colloid Interface Sci.

[81] David R, Joergen F, Steven F, Michael H, Stefanie H, Francesco L (2014). Hypersensitivity reactions to intravenous iron: guidance for risk minimization and management. Haematologica.

[82] Moghimi SM, Simberg D (2017). Complement activation turnover on surfaces of nanoparticles. Nano Today.

[83] Goram AL, Richmond PL (2001). Pegylated Liposomal Doxorubicin: Tolerability and Toxicity. Pharmacotherapy. J Human Pharmacology and Drug Therapy.

[84] Onwukwe C, Maisha N, Holland M, Varley M, Groynom R, Hickman D (2018). Engineering Intravenously Administered Nanoparticles to Reduce Infusion Reaction and Stop Bleeding in a Large Animal Model of Trauma. Bioconjug Chem.

[85] Ricklin D, Lambris JD (2013). Complement in Immune and Inflammatory Disorders: Pathophysiological Mechanisms. J Immunol.

[86] Maisha N, Rubenstein M, Bieberich CJ, Lavik E (2021). Getting to the Core of It All: Nanocapsules to Mitigate Infusion Reactions Can Promote Hemostasis and Be a Platform for Intravenous Therapies. Nano Lett.

[87] Chen C, Li H, Pan J, Yan Z, Yao Z, Fan W (2015). Biodegradable composite scaffolds of bioactive glass/chitosan/carboxymethyl cellulose for hemostatic and bone regeneration. Biotechnol Lett.

[88] Zhu J-q, Zhang W-g, Zhang Y-q, Tang H (2020). Swelling characteristics of East-Africa black cotton soil based on computer molecular simulation. J Cent South Univ.

[89] Gentile F, Chiappini C, Fine D, Bhavane RC, Peluccio MS, Cheng MM-C (2008). The effect of shape on the margination dynamics of non-neutrally buoyant particles in two-dimensional shear flows. J Biomech.

[90] Girish A, Sekhon U, Sen Gupta A (2020). Bioinspired artificial platelets for transfusion applications in traumatic hemorrhage. Transfusion.

[91] Gao Y, Sarode A, Kokoroskos N, Ukidve A, Zhao Z, Guo S (2020). A polymer-based systemic hemostatic agent. Sci Adv.

[92] Jones CF, Campbell RA, Brooks AE, Assemi S, Tadjiki S, Thiagarajan G (2012). Cationic PAMAM Dendrimers Aggressively Initiate Blood Clot Formation. ACS Nano.

[93] Cheng J, Feng S, Han S, Zhang X, Chen Y, Zhou X (2016). Facile Assembly of Cost-Effective and Locally Applicable or Injectable Nanohemostats for Hemorrhage Control. ACS Nano.

[94] Tu H, Yu Y, Chen J, Shi X, Zhou J, Deng H (2017). Highly cost-effective and high-strength hydrogels as dye adsorbents from natural polymers: chitosan and cellulose. Polym Chem.

[95] Wang H, Qian J, Ding F (2018). Emerging Chitosan-Based Films for Food Packaging Applications. J Agric Food Chem.

[96] Gopalakrishnan L, Ramana LN, Sethuraman S, Krishnan UM (2014). Ellagic acid encapsulated chitosan nanoparticles as anti-hemorrhagic agent. Carbohydr Polym.

[97] T C, P L, S W, Y C (2014). Adenosine diphosphate-decorated chitosan nanoparticles shorten blood clotting times, influencing the structures and varying the mechanical properties of the clots. Int J Nanomedicine.

[98] Zhang P, Li S, Zhang S, Zhang X, Wan L, Yun Z (2018). GRGDS-functionalized chitosan nanoparticles as a potential intravenous hemostat for traumatic hemorrhage control in an animal model. Nanomed Nanotechnol Biol Med.

[99] Bertram JP, Williams CA, Robinson R, Segal SS, Flynn NT, Lavik EB (2009). Intravenous Hemostat: Nanotechnology to Halt Bleeding. Sci Transl Med.

[100] Lashof-Sullivan M, Holland M, Groynom R, Campbell D, Shoffstall A, Lavik E (2016). Hemostatic Nanoparticles Improve Survival Following Blunt Trauma Even after 1 Week Incubation at 50 °C. ACS Biomater Sci Eng.

[101] Kang N, Perron M-È, Prud'homme RE, Zhang Y, Gaucher G, Leroux J-C (2005). Stereocomplex Block Copolymer Micelles: Core-Shell Nanostructures with Enhanced Stability. Nano Lett.

[102] Yu M, Wang J, Yang Y, Zhu C, Su Q, Guo S (2016). Rotation-Facilitated Rapid Transport of Nanorods in Mucosal Tissues. Nano Lett.

[103] Tao L, Hu W, Liu Y, Huang G, Sumer BD, Gao J (2011). Shape-specific polymeric nanomedicine: emerging opportunities and challenges. Exp Biol Med.

[104] Pillai JD, Dunn SS, Napier ME, DeSimone JM (2011). Novel platforms for vascular carriers with controlled geometry. IUBMB Life.

[105] He Y, Xu J, Sun X, Ren X, Maharjan A, York P (2019). Cuboidal tethered cyclodextrin frameworks tailored for hemostasis and injured vessel targeting. Theranostics.

[106] Duarte AP, Coelho JF, Bordado JC, Cidade MT, Gil MH (2012). Surgical adhesives: Systematic review of the main types and development forecast. Prog Polym Sci.

[107] di Lena F (2014). Hemostatic polymers: the concept, state of the art and perspectives. J Mater Chem B.

[108] Levy JH, Szlam F, Tanaka KA, Sniecienski RM (2012). Fibrinogen and Hemostasis: A Primary Hemostatic Target for the Management of Acquired Bleeding. Anesth Analg.

[109] Spahn DR, Bouillon B, Cerny V, Coats TJ, Duranteau J, Fernández-Mondéjar E (2013). Management of bleeding and coagulopathy following major trauma: an updated European guideline. Crit Care.

[110] Roberts I (2015). Tranexamic acid in trauma: how should we use it?. J Thromb Haemost.

[111] Chan LW, Wang X, Wei H, Pozzo LD, White NJ, Pun SH (2015). A synthetic fibrin cross-linking polymer for modulating clot properties and inducing hemostasis. Sci Transl Med.

[112] Chan LW, White NJ, Pun SH (2016). A Fibrin Cross-linking Polymer Enhances Clot Formation Similar to Factor Concentrates and Tranexamic Acid in an in Vitro Model of Coagulopathy. ACS Biomater Sci Eng.

[113] Muszbek L, Bereczky Z, Bagoly Z, Komáromi I, Katona É (2011). Factor XIII: A Coagulation Factor With Multiple Plasmatic and Cellular Functions. Physiol Rev.

[114] Hun Yeon J, Chan KYT, Wong T-C, Chan K, Sutherland MR, Ismagilov RF (2015). A biochemical network can control formation of a synthetic material by sensing numerous specific stimuli. Sci Rep.

[115] Milleret V, Simona BR, Lienemann PS, Vörös J, Ehrbar M (2014). Electrochemical Control of the Enzymatic Polymerization of PEG Hydrogels: Formation of Spatially Controlled Biological Microenvironments. Adv Healthc Mater.

[116] Hart RG, Diener H-C, Coutts SB, Easton JD, Granger CB, O'Donnell MJ (2014). Embolic strokes of undetermined source: the case for a new clinical construct. Lancet Neurol.

[117] Zhao H, Ma L, Gao C, Shen J (2009). A composite scaffold of PLGA microspheres/fibrin gel for cartilage tissue engineering: Fabrication, physical properties, and cell responsiveness. J Biomed Mater Res B.

[118] Chan KYT, Zhao C, Siren EMJ, Chan JCY, Boschman J, Kastrup CJ (2016). Adhesion of Blood Clots Can Be Enhanced When Copolymerized with a Macromer That Is Crosslinked by Coagulation Factor XIIIa. Biomacromolecules.

[119] Gillespie AH, Doctor A (2021). Red Blood Cell Contribution to Hemostasis. Front Pediatr.

[120] Zhu Z, Zhang K, Xian Y, He G, Pan Z, Wang H (2023). A Choline Phosphoryl-Conjugated Chitosan/Oxidized Dextran Injectable Self-Healing Hydrogel for Improved Hemostatic Efficacy. Biomacromolecules.

[121] Taboada GM, Yang K, Pereira MJN, Liu SS, Hu Y, Karp JM (2020). Overcoming the translational barriers of tissue adhesives. Nat Rev Mater.

[122] Hu H, Xu F-J (2020). Rational design and latest advances of polysaccharide-based hydrogels for wound healing. Biomater Sci.

[123] Dong R, Guo B (2021). Smart wound dressings for wound healing. Nano Today.

[124] Jiang S, Liu S, Lau S, Li J (2022). Hemostatic biomaterials to halt non-compressible hemorrhage. J Mater Chem B.

[125] Auriemma M, Piscitelli A, Pasquino R, Cerruti P, Malinconico M, Grizzuti N (2015). Blending poly(3-hydroxybutyrate) with tannic acid: Influence of a polyphenolic natural additive on the rheological and thermal behavior. Eur Polym J.

[126] Liu W, Ou-Yang W, Zhang C, Wang Q, Pan X, Huang P (2020). Synthetic Polymeric Antibacterial Hydrogel for Methicillin-Resistant Staphylococcus aureus-Infected Wound Healing: Nanoantimicrobial Self-Assembly, Drug- and Cytokine-Free Strategy. ACS Nano.

[127] Guo S, Ren Y, Chang R, He Y, Zhang D, Guan F (2022). Injectable Self-Healing Adhesive Chitosan Hydrogel with Antioxidative, Antibacterial, and Hemostatic Activities for Rapid Hemostasis and Skin Wound Healing. ACS Appl Mater Interfaces.

[128] Yang L, Wang H (2023). Relationships between pretreatment methods and properties of ion-electroactive polymers based on image analysis technology. Tribol Int.

[129] Wang H, Yang L (2023). Effects of effective voltages, electrode types and stretching states on electrical properties and actuation characteristics of dielectric elastomer materials. Polym Test.

[130] Yang L, Yang Y, Wang H (2023). Modeling and control of ionic polymer metal composite actuators: A review. Eur Polym J.

[131] Spotnitz WD (2012). Article Commentary: Hemostats, Sealants, and Adhesives: A Practical Guide for the Surgeon. The American Surgeon™.

[132] Chen G, Yu Y, Wu X, Wang G, Ren J, Zhao Y (2018). Bioinspired Multifunctional Hybrid Hydrogel Promotes Wound Healing. Adv Funct Mater.

[133] Lokhande G, Carrow JK, Thakur T, Xavier JR, Parani M, Bayless KJ (2018). Nanoengineered injectable hydrogels for wound healing application. Acta Biomater.

[134] Huang Y, Zhao X, Zhang Z, Liang Y, Yin Z, Chen B (2020). Degradable Gelatin-Based IPN Cryogel Hemostat for Rapidly Stopping Deep Noncompressible Hemorrhage and Simultaneously Improving Wound Healing. Chem Mater.

[135] Huang H, Chen H, Wang X, Qiu F, Liu H, Lu J (2019). Degradable and Bioadhesive Alginate-Based Composites: An Effective Hemostatic Agent. ACS Biomater Sci Eng.

[136] Simpson A, Shukla A, Brown AC (2022). Biomaterials for Hemostasis. Annu Rev Biomed Eng.

[137] Johnson L, Tan S, Wood B, Davis A, Marks DC (2016). Refrigeration and cryopreservation of platelets differentially affect platelet metabolism and function: a comparison with conventional platelet storage conditions. Transfusion.

[138] Liu C, Liu C, Yu S, Wang N, Yao W, Liu X (2020). Efficient antibacterial dextran-montmorillonite composite sponge for rapid hemostasis with wound healing. Int J Biol Macromol.

[139] Han W, Zhou B, Yang K, Xiong X, Luan S, Wang Y (2020). Biofilm-inspired adhesive and antibacterial hydrogel with tough tissue integration performance for sealing hemostasis and wound healing. Bioact Mater.

[140] Wu Y-K, Cheng N-C, Cheng C-M (2019). Biofilms in Chronic Wounds: Pathogenesis and Diagnosis. Trends Biotechnol.

[141] Zhu J, Li F, Wang X, Yu J, Wu D (2018). Hyaluronic Acid and Polyethylene Glycol Hybrid Hydrogel Encapsulating Nanogel with Hemostasis and Sustainable Antibacterial Property for Wound Healing. ACS Appl Mater Interfaces.

[142] Chee E, Nandi S, Nellenbach K, Mihalko E, Snider DB, Morrill L (2020). Nanosilver composite pNIPAm microgels for the development of antimicrobial platelet-like particles. J Biomed Mater Res B.

[143] Sproul EP, Nandi S, Chee E, Sivadanam S, Igo BJ, Schreck L (2020). Development of Biomimetic Antimicrobial Platelet-Like Particles Comprised of Microgel Nanogold Composites. Regen Eng Transl Med.

[144] Bartlett TR, Sokolov SV, Plowman BJ, Young NP, Compton RG (2016). Tracking of photochemical Ostwald ripening of nanoparticles through voltammetric atom counting. Nanoscale.

[145] Kharissova OV, Dias HVR, Kharisov BI, Pérez BO, Pérez VMJ (2013). The greener synthesis of nanoparticles. Trends Biotechnol.

